# Synthetic circRNA therapeutics: innovations, strategies, and future horizons

**DOI:** 10.1002/mco2.720

**Published:** 2024-11-09

**Authors:** Jingsheng Cai, Zonghao Qiu, William Chi‐Shing Cho, Zheng Liu, Shaoyi Chen, Haoran Li, Kezhong Chen, Yun Li, Chijian Zuo, Mantang Qiu

**Affiliations:** ^1^ Thoracic Oncology Institute & Research Unit of Intelligence Diagnosis and Treatment in Early Non‐Small Cell Lung Cancer Peking University People's Hospital Beijing China; ^2^ Department of Thoracic Surgery Peking University People's Hospital Beijing China; ^3^ Institute of Advanced Clinical Medicine Peking University Beijing China; ^4^ Suzhou CureMed Biopharma Technology Co., Ltd. Suzhou China; ^5^ Department of Clinical Oncology Queen Elizabeth Hospital Hong Kong China

**Keywords:** circRNA vaccine, group I intron, internal ribosome entry site, lipid nanoparticle, synthetic circRNA

## Abstract

Small molecule drugs are increasingly emerging as innovative and effective treatments for various diseases, with mRNA therapeutics being a notable representative. The success of COVID‐19 vaccines has underscored the transformative potential of mRNA in RNA therapeutics. Within the RNA family, there is another unique type known as circRNA. This single‐stranded closed‐loop RNA molecule offers notable advantages over mRNA, including enhanced stability and prolonged protein expression, which may significantly impact therapeutic strategies. Furthermore, circRNA plays a pivotal role in the pathogenesis of various diseases, such as cancers, autoimmune disorders, and cardiovascular diseases, making it a promising clinical intervention target. Despite these benefits, the application of circRNA in clinical settings remains underexplored. This review provides a comprehensive overview of the current state of synthetic circRNA therapeutics, focusing on its synthesis, optimization, delivery, and diverse applications. It also addresses the challenges impeding the advancement of circRNA therapeutics from bench to bedside. By summarizing these aspects, the review aims to equip researchers with insights into the ongoing developments and future directions in circRNA therapeutics. Highlighting both the progress and the existing gaps in circRNA research, this review offers valuable perspectives for advancing the field and guiding future investigations.

## Graphical abstract

This pie chart visually represents the comprehensive content of this review, categorized into four main sections: circRNA synthesis, circRNA optimizations, delivery platforms, and circRNA therapeutics. Each section is further divided into specific subtopics, facilitating a quick and clear understanding of the key points covered in this review. circRNA, circular RNA

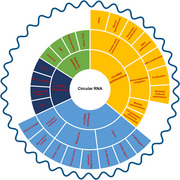

## 1 INTRODUCTION

Circular RNA (circRNA), a unique subtype within the RNA family, comprises a covalently closed single‐strand structure.^1^ It was originally discovered in plant viroids in 1976.^2^ Since then, circRNA has been observed in eukaryotic cells,^3^ yeast mitochondria,^4^ Tetrahymena,^5^ hepatitis delta (δ) virus,^6^ human E‐twenty‐six‐1 gene,^7^ and so on. In recent years, high‐throughput next‐generation sequencing techniques and powerful bioinformatics tools have aided in the identification of diverse circRNAs across multiple organisms.^1^, ^8^, ^9^, ^10^ circRNA could be roughly classified into exonic circRNA,^11^ intronic circRNA,^12^ exon–intron circRNA,^13^ fusion circRNA,^14^ readthrough circRNA,^15^ and tRNA‐derived circRNA.^16^ Most circRNAs are generated by pre‐messenger RNA (mRNA) back splicing, where a downstream 5′ donor splice site connects to an upstream 3′ acceptor splice site, forming a 3′–5′phosphodiester bond and yielding an RNA in a circular format.^11^, ^17^, ^18^, ^19^, ^20^ There are several mechanisms reported to be involved in endogenous circRNA formation including intron‐pairing‐mediated circularization (direct back‐splicing),^21^ RNA‐binding protein‐mediated circulation,^22^ and lariat‐driven circularization (exon skipping).^23^

It has long been held that circRNA is a by‐product of pre‐mRNA mis‐splicing and possesses noncoding functionalities such as transcription modulation,^24^ splicing regulation,^25^ scaffold for protein complex,^26^ immune response modulator,^27^, ^28^ signal transducing,^29^ and molecular sponges.^25^, ^30^ These regulatory roles allow circRNA to significantly contribute to the pathogenesis of numerous diseases. Extensive research has highlighted the oncogenic regulatory roles of circRNA in cancer progression. For instance, our team identified a proto‐oncogenic circRNA, circPRKCI, from the 3q26.2 amplicon in lung adenocarcinoma, which is overexpressed due to locus amplification and promotes tumorigenesis by sponging miR‐545 and miR‐589.^31^ Other colleagues at our department have discovered silencing circRNA HIPK3 (circHIPK3) impaired cell proliferation, migration, invasion, and induced autophagy via the MIR124‐3p–STAT3–PRKAA/AMPKa axis in STK11 mutant lung cancer cell lines.^32^ In addition to cancer, circRNA plays vital roles in other diseases. For example, extracellular vesicles containing circCDK13 accelerate wound healing in preclinical diabetic models^33^; the absence of circAnk3 in the mouse hippocampus results in anxiety‐like behavior and social deficits through the miR‐7080‐3p/IQGAP1 pathway^34^; and statins improve cardiac endothelial function, protect ejection fraction, and prevent heart failure by upregulating circRNA–RBCK1 expression.^35^ These studies suggest that untranslated circRNA plays a crucial role in the onset and progression of diseases, making it a potentially significant target for therapeutic intervention. Recent studies have also uncovered its translational potential, which operates independently of the cap‐dependent mechanism. One example is the presence of an internal ribosomal entry site (IRES) sequence within circRNA, enabling it to directly recruit ribosomes and initiate translation.^36^ Additionally, N^6^‐methyladenosine (m^6^A) modification on endogenous circRNA can induce translation by recruiting the eukaryotic translation initiation factor 4 gamma 2 and the YTH N6‐methyladenosine RNA Binding Protein F3 m^6^A reader.^37^, ^38^ These translatable circRNAs that encode proteins have been detected in various human tissues and may be associated with a range of human diseases including cancer.^39^, ^40^ Consequently, specific pathogenic translatable circRNAs have become attractive targets for therapeutic intervention in disease progression.^41^

In addition to endogenous circRNA as mentioned above, synthetic circRNA has also become a focal point of interest for many researchers. With the growing comprehension of RNA structure and function, coupled with advancements in nucleic acid synthesis technology, there has been a remarkable rise in the in vitro synthesis of translation‐competent RNAs.^42^, ^43^, ^44^ This innovative approach enables the precise expression of specific proteins within designated cells, tissues, and organs, aiming to effectively treat certain diseases. Notably, mRNA‐based COVID‐19 vaccines have emerged as one of the most prominent applications of this approach.^45^, ^46^ Recently, despite the current surge in mRNA therapeutics development and the numerous pipelines under investigation, many related issues have surfaced that urgently need to be addressed. For example, the inherent instability of mRNA poses high requirements for production, transportation, and storage condition.^47^, ^48^ Additionally, the short half‐life of mRNA leads to a short duration of target protein expression, necessitating repeated administration.^49^, ^50^, ^51^ In this context, researchers have shifted their focus to an alternative format of RNA template‐circRNA. Compared with mRNA, circRNA possesses a unique circular structure that confers resistance against exonucleases, making it more stable.^1^ Literature have reported that the half‐life of circRNA is approximately 2.5 times longer than that of mRNA, significantly enhancing the duration of protein expression.^52^, ^53^ It appears that circRNA may serve as a potential 2.0 version of mRNA, with the potential to revolutionize molecular therapy in the future by enabling the development of circRNA‐based small molecule drugs. However, circRNA therapeutics are currently in their infancy, with numerous unknowns and limitations that require further exploration and optimization.

The RNA therapeutics industry is rapidly advancing, positioning RNA as the next generation of blockbuster small molecule drugs. circRNA therapy is emerging as a rising star within this landscape. With its unique advantages of efficient synthesis and stable high expression, circRNA has already demonstrated potential in vaccines, immunotherapy, and protein replacement therapies, promising innovative and effective treatment options for human diseases.^54^, ^55^, ^56^ However, circRNA therapies currently still face limitations and unknowns. It is essential to summarize the current progress in circRNA synthesis and applications, as well as the challenges that need to be addressed. This review aims to offer a comprehensive overview of in vitro circRNA synthesis, including chemical synthesis, ligase synthesis, and ribozyme synthesis; circRNA optimization to enhance protein expression, reduce immunogenicity, improve circularization, and increase stability; delivery platform for circRNA, including virus vectors, electroporation, lipid nanoparticle (LNP) and exosome; clinical applications of circRNA therapeutics, including COVID‐19 and other vaccines, gene expression regulation, and adoptive cell therapies in hematological malignancies and other solid tumors; as well as the associated challenges and prospects. We aim to provide researchers with insights into the ongoing developments and future directions in circRNA therapeutics by summarizing these aspects. By emphasizing both the progress made and the existing gaps in circRNA research, it offers valuable perspectives to advance the field and guide future investigations.

## 2 IN VITRO circRNA SYNTHESIS

**FIGURE 1:**
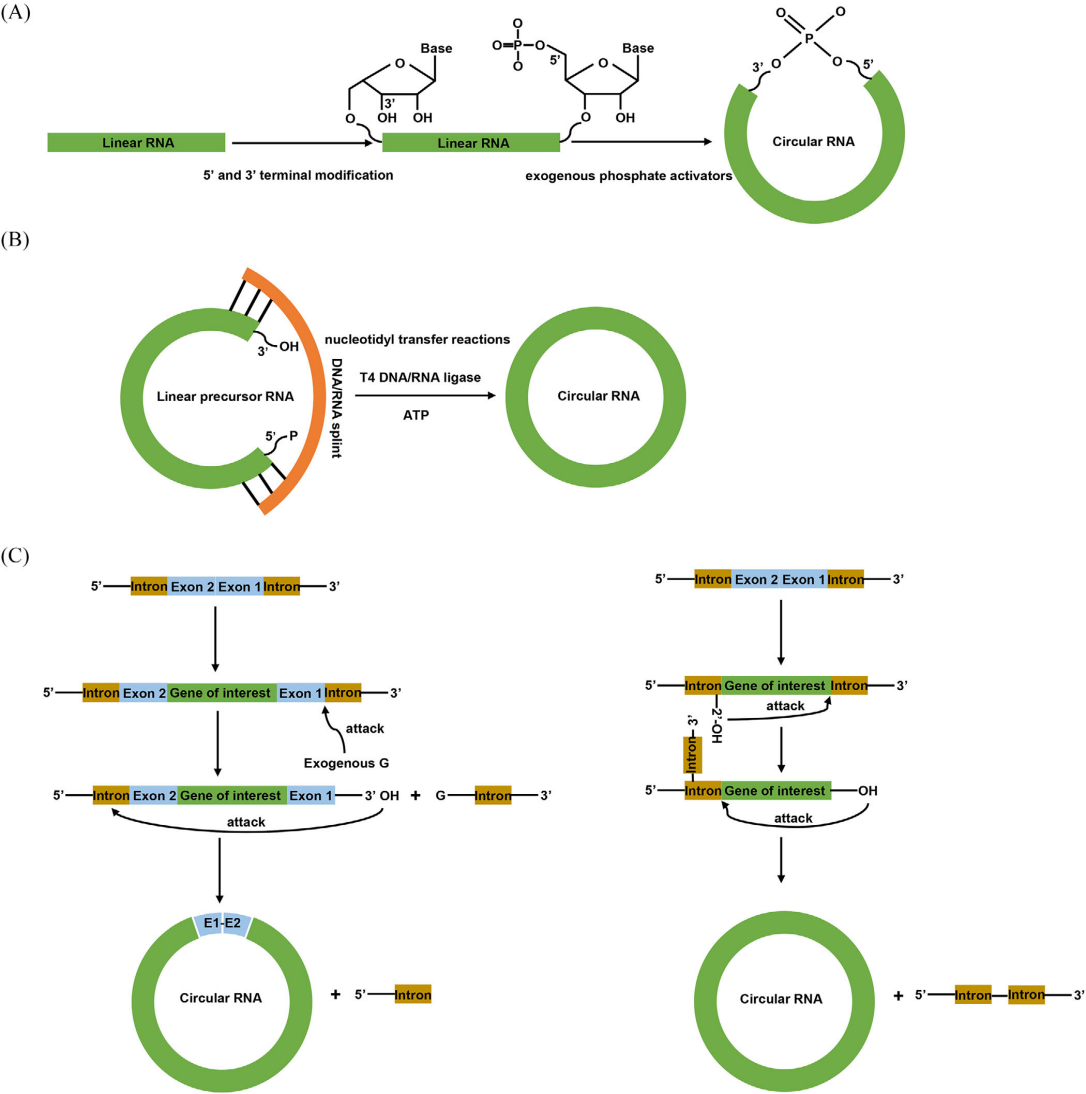
In vitro circRNA synthesis. (A) Chemical synthesis. Linear RNA molecule is initially modified with 5′‐P and 3′‐OH groups. Subsequently, the 5′ terminal can be ligated with 3′ terminal to form phosphodiester bond with the assistance of exogenous phosphate activators; (B) T4 enzymatic ligation. Linear RNA molecule containing 5′‐P and 3′‐OH groups can be ligated through a nucleotidyl transfer reaction with the support of T4 DNA/RNA ligase, ATP and DNA/RNA splint; (C) ribozymes ligation. Left: group I intron self‐splicing system, in the first transesterification reaction, the 5′‐half intron of the construct is released in the presence of exogenous G. In the second transesterification reaction, the newly generated free 3′‐OH group of the 3′‐half exon attacks the 3′‐splice site, ultimately resulting in the formation of circRNA and the release of the 3′‐half intron; right: group II intron self‐splicing system, the self‐splicing process is initiated by a nucleophilic attack of the 2′‐OH group of the branch‐point adenosine residue on the 5′ splice site, leading to the formation of a branched intermediate structure. Subsequently, the 3′ splice site is attacked by the free 3′‐OH group of the exon sequence, resulting in the excision of the intron and ligation of the flanking exons to produce a circRNA molecule. 5′‐P, 5′‐phosphate; 3′‐OH, 3′‐hydroxyl; 2′‐OH, 2′‐hydroxyl; G, guanine; circRNA, circular RNA.

In order to achieve the large‐scale synthesis of circRNA, researchers have conducted a series of explorations. Currently, there are three main methods for in vitro circRNA synthesis including chemical synthesis,^57^ enzymatic synthesis based on DNA or RNA ligases,^58^ and ribozyme‐based methods relying on the self‐splicing introns^59^, ^60^ (Figure 1 and Table 1). Each of these three methods possesses its own characteristics, advantages and limitations.

**TABLE 1:** Comparative analysis of three synthetic methods for circRNA.

Characteristics	Chemical synthesis	T4 enzymatic ligation	Ribozyme ligation
Synthesis^61^, ^62^, ^63^, ^64^			
Principle	Uses chemical reagents to link RNA molecules with functionalized ends	Uses DNA or RNA ligases to catalyze the reactions	Employs self‐catalytic ribozyme sequences for self‐splicing and ligation
Materials	End modification reagents (e.g., phosphates, thiols, alkynes, or amines) and catalytic chemical reagents	Ligases, ATP, splint	Self‐splicing introns
Linkage	Ligation via phosphodiester bonds or non‐natural linkages	Enzymes catalyze nucleotidyl transfer reactions to form phosphodiester bonds	Involves two transesterification reactions and forms phosphodiester bonds
Advantages^58^, ^65^, ^66^	Effective for synthesis of small circRNAs; the system is relatively mature	Effective for synthesizing circRNAs with moderate length; direct and efficient ligation of RNA termini; high ligation efficiency with splints	Suitable for synthesizing circRNAs of various sizes (up to 5 kb); simple reaction conditions; High ligation efficiency
Limitations^58^, ^65^, ^66^	Low yield but with high purification cost; typically synthesizes RNAs less than 50–70 nucleotides; Risk of toxic reagents; potential for intermolecular ligations and possible formation of 2′–5′ non‐natural phosphodiester bonds	Reduced efficiency with longer or structured RNA; intermolecular ligations; potential introduction of exogenous nucleotides	Potential for introducing scar sequences; requires specific sequence contexts; production of several splicing intermediate byproducts
Reaction efficiency^63^	Low efficiency	High efficiency	High efficiency
Yield and purity^57^, ^67^	Yields range from low to moderate; high purity achievable with click reactions	High yield; can introduce 2–3 exogenous nucleotides; high purity generally achievable, especially with T4 RNA ligase I	High yield; potential for introducing scar sequences; high purity in well‐optimized systems like Clean‐PIE
Scalability^57^	Challenging due to complexity and cost; suitable for smaller‐scale applications or specialized purposes	More scalable than chemical synthesis; suitable for medium‐scale applications	Highly scalable for large RNA circles; suitable for large‐scale production
Safety considerations^58^, ^65^	Requires handling of toxic reagents like BrCN; potential biosafety issues due to non‐natural phosphodiester bonds	Generally safe; no significant biosafety issues, but incomplete reactions may lead to by‐products	Minimal safety concerns; ribozyme‐based methods generally involve nonhazardous reagents
Cost^67^	High due to the need for specialized reagents and equipment, low yields, and extensive purification steps	Moderate; cost effective for long RNA synthesis.	Generally lower cost compared with chemical synthesis; high efficiency can offset initial setup costs
Optimization needs^58^, ^68^	Requires careful design of linkers and functional groups to ensure high yield and purity; optimization of reaction conditions critical	Pre‐orientation of RNA ends necessary to avoid multimer formation; optimization of splints and reaction conditions critical	Optimization of sequence contexts and ribozyme elements essential; requires careful design to avoid scar sequences

Abbreviations: circRNA, circular RNA; BrCN, cyanogen bromide; PIE, permuted intron–exon.

### 2.1 Chemical methods for circRNA synthesis

The initial step in circRNA preparations involves the synthesis of linear RNA molecules with appropriate 5′ and 3′ terminal functionalities for subsequent ligation.^57^ For chemical circRNA synthesis, 5′ terminal and 3′ terminal are modified with phosphates, thiols, alkynes, or amines, which facilitate end joining.^69^ After that, the 5′ phosphate (5′‐P) can be ligated with the 3′hydroxyl (3′‐OH) or the 5′ hydroxyl (5′‐OH) can be ligated with the 3′ phosphate (3′‐P), using exogenous phosphate activators such as cyanogen bromide (BrCN) or water‐soluble ethyl‐3‐(30‐dimethylaminopropyl)‐carbodiimide (EDC), to form phosphodiester bonds^57^ (Figure 1A). In addition to utilizing natural phosphodiester linkages formed by phosphoric acid activators, other chemical methods can be employed to create non‐natural linkages such as the oxime linkage, triazole linkage and thioether linkage.^70^, ^71^ By connecting the two ends using these non‐natural linkages, the linear RNA precursor can be circularized into circRNA. For example, click chemistry‐based circRNA synthesis strategies have emerged as powerful methods for the efficient formation of circRNA molecules by covalently connecting the functionalized ends of a linear RNA through a simple click reaction.^57^, ^72^

Although chemical synthesis has been extensively employed and continuously refined for over a decade, it still faces significant challenges.^73^, ^74^ (1) One major challenge is the efficient and selective circularization of linear RNA. While click circularization techniques have shown promise, they still need optimizations to ensure high purity and yields of circRNA products. (2) Another challenge lies in site‐specific modifications and functionalization of circRNA. Current chemical synthetic approaches often lack the selectivity and precision required for site‐specific modifications, leading to heterogeneous products. (3) The formation of intermolecular or intramolecular multimers poses a great challenge during the chemical synthetic process. (4) The chemical synthesis methods also entail certain safety risks, exemplified by toxic and hazardous chemical reagents such as BrCN and EDC. Eliminating these chemical agents represents a distinct challenge. (5) The scalability and cost effectiveness of chemical circRNA synthesis still need improvements. Current synthetic approaches often suffer from low yields, limited oligomers length (50–70 nucleotides), long reaction time, and high costs. Therefore, searching more efficient and economical synthetic strategies is essential for advancing chemical synthetic circRNA research and translation to clinical applications.

### 2.2 T4 enzymatic ligation in circRNA synthesis

Enzymatic ligation methods utilizing DNA or RNA ligases have been widely employed in the synthesis of circRNA. These methods involve the use of enzymes such as T4 DNA ligase, T4 RNA ligase I, and T4 RNA ligase II. These ligases could directly connect RNA termini possessing a 5′‐P group and a 3′‐OH group through nucleotidyl transfer reactions^75^ (Figure 1B). To be more specific^57^: (1) the enzyme initiates a reaction with ATP and form a covalent intermediate known as ligase‐AMP; (2) the AMP component of the intermediate is then transferred to the RNA terminus with a 5′‐P, resulting in the formation of a 5′‐RNA‐adenylate called AppRNA; (3) finally, the AppRNA undergoes attack by the 3′‐OH group, leading to the formation of a phosphodiester bond.

#### 2.2.1 T4 DNA ligase‐mediated circRNA synthesis

T4 DNA ligase, an enzyme derived from the bacteriophage T4, was initially recognized for its ability to join DNA strands in the late 1970s.^76^ It could catalyze the formation of a phosphodiester bond by ligating a 5′‐P group with a 3′‐OH group of two DNA fragments with the help of ATP cofactor and a splint.^77^ Although T4 DNA ligase is commonly used for DNA fragments ligation, it is less frequently employed for the connection of RNA fragments.^78^ In the late 20th century, DNA ligase I was utilized for the circularization of RNA. During this time, Moore and colleagues^79^ have developed a method that involved ligating distinct RNA fragments to synthesize long‐chain RNA with specific modifications. Subsequently, Chen et al.^36^ demonstrated that circRNA derived from a 453‐nucleotide pre‐mRNA using T4 DNA ligase, could be translated into proteins. In recent years, researchers have discovered that a circular dumbbell RNA/DNA chimeric oligonucleotides, synthesized using T4 DNA ligase, has the ability to inhibit HIV‐1 replication.^80^

#### 2.2.2 T4 RNA ligase 1‐mediated circRNA synthesis

T4 RNA ligase 1, originating from bacteriophage T4, was first reported in 1962. This enzyme also facilitates the joining of a 5′‐P‐terminated nucleic acid donor with a 3′‐OH‐terminated nucleic acid acceptor through the formation of a phosphodiester bond, coupled with the hydrolysis of ATP to AMP and pyrophosphoric acid.^81^ During synthesis, the presence of the RNA intrinsic secondary structure or a complementary splint that hybridizes with both the termini of the donor and acceptor nucleic acids can significantly enhance the ligation efficiency.^57^, ^82^, ^83^ Although the use of a splint can increase the circularization efficiency, it may also introduce two to three exogenous nucleotides during this process.^82^ T4 RNA ligase 1 plays a vital role in RNA ligation and exhibits remarkable effectiveness in ligating small fragments of RNA molecules.^57^ For instance, Chen et al.^84^ have generated circRNAs using T4 RNA ligase 1, resulting in products that are less immunogenic but more stable compared with those generated using ribozyme ligation methods.

#### 2.2.3 T4 RNA ligase 2‐mediated circRNA synthesis

T4 RNA ligase 2, similar to T4 RNA ligase 1, catalyzes the ligation of RNA acceptor with a 3′‐OH end and RNA donor with a 5′‐P group end using ATP as a cofactor. This enzyme was discovered by Ho et al. in 2002.^85^ T4 RNA ligase 2 exhibits a broad substrate specificity and can efficiently ligate RNA fragments with various end modifications.^85^ It belongs to a widely distributed family of RNA ligases, sharing a significant sequence similarity with DNA ligases.^86^ In contrast, RNA ligase 1, a poor prototype, exhibits limited sequence similarity with DNA ligases.^87^ T4 RNA ligase 2 demonstrates enhanced efficiency in ligating noncomplementary RNA molecules compared with T4 RNA ligase 1.^88^ However, T4 RNA ligase 2 may show reduced efficiency when ligating longer or more structured RNA molecules, and it can introduce incorrect RNA‐end connections, resulting in the production of noncircular products.^85^

### 2.3 Ribozymes ligation for circRNA synthesis

Considering that chemical synthesis and T4 enzymatic ligation methods are commonly used for producing smaller‐sized RNA circles, there is a need for alternative methods to generate larger circRNAs.^57^ Historically, proteins are regarded as the only molecules possessing catalytic functions until 1980s, when scientists discovered that the 26S ribosomal RNA of Tetrahymena thermophila could undergo autoexcision and autocyclization.^89^ To differentiate them from protein enzymes, biologically active RNA molecules with catalytic functions are collectively referred to as ribozymes.^90^ There are several ribozymes‐based circRNA synthesis methods, and the most prominent one is permuted introns and exons (PIE) method.

#### 2.3.1 Group I intron‐mediated circRNA synthesis

The PIE circularization strategy, which is commonly favored by group I intron self‐splicing systems, finds extensive use in the synthesis of circRNA. The exact circularization mechanism of PIE involves two transesterification reactions (Figure 1C, left).^91^ During the first transesterification reaction, the 3′‐terminal sequence (5′‐half intron) of the PIE construct is released in the presence of exogenous guanosine and Mg^+^. In the second transesterification reaction, the newly generated free 3′‐OH group of the 3′‐half exon attacks the 3′‐splice site. This ultimately leads to the formation of circRNA and the release of the 3′‐half intron. PIE was first reported in 1992 when Puttaraju and Been^91^ discovered that permuted group I intron precursor RNAs derived from Anabena pre‐tRNA could self‐splice and generate circRNA exons in vitro. After 2 years, Ford and Ares^92^ further optimized the method by inserting foreign sequences into the exons of a group I intron from the thymidylate synthase (td) gene of phage T4. Since then, the PIE elements from Anabaena pre‐tRNA or T4 td genes have emerged as fundamental backbones in circularizing a diverse array of target sequences inserted between the group I introns.^93^, ^94^, ^95^

A pivotal milestone event occurred in 2018 when Wesselhoeft et al.^43^ made substantial improvements to the existing PIE circularization structure. Building upon this breakthrough technology, they established oRNA Therapeutics, specializing in circRNA‐based treatments. By incorporating homology arms and spacer elements, Wesselhoeft et al.^43^ successfully increased the efficiency of circularization. Additionally, their research revealed that the Anabaena PIE system outperforms the T4 phage PIE system in terms of both splicing efficiency and circRNA production.^43^ However, the oRNA PIE method still has some limitations. For instance, it is unable to remove exon1–exon2 sequences (E1–E2) and spacer (both are also referred to as “scar sequence”) that aid in the linear RNA circularization process, and these exogenous sequences can contribute to immunogenicity of circular products.^84^ To address this limitation, researchers have explored several improved methods. In 2021, Rausch et al.^96^ proposed, for the first time, to identify suitable cleavage sites within open reading frames (ORFs) that result in end sequences similar to E1–E2 (candidate E1–E2 homologs), thereby promoting circRNA synthesis as a substitute for E1–E2. Furthermore, they have conducted serial mutagenesis on the E1–E2 sequences to investigate the impact of E1–E2 sequence variations on RNA circularization.^96^ As a result, they have provided guidelines for designing custom‐tailored PIE transcription templates, which enable efficient circRNA synthesis of almost any sequence.^96^ In the following year, our research team has developed a similar PIE method called “Clean‐PIE.”^68^ In this method, circRNA can be synthesized through the optimized group I ribozymatic reaction‐mediated ligation of the flanking segment sequences that are concealed within the IRES or CDS.^68^ We have also developed an algorithm, called “nucleotide‐replacement” system, to select the most appropriate splicing site within the ORF, and the terminals of the splinted sequence matched E1–E2 sequence almost perfectly, facilitating the circularization of the target fragments.^68^ Both of these methods could reduce the immunogenicity of PIE‐based circRNA. In addition, Lee et al.^97^ have developed a novel circRNA synthesis method using autocatalytical end‐to‐end self‐targeting and splicing (STS) reaction based on Tetrahymena group I intron ribozyme. Their findings indicated that circRNA synthesis using STS showed comparable efficacy to that of PIE, but it barely triggers innate immune reactions.^97^ However, these findings are primarily dependent on the concurrent inclusion of poly(I:C), and the diverse purification methods employed may introduce variability in the immunogenicity of circRNA. Hence, the effectiveness of this synthetic approach necessitates further evaluation. Similarly, a Chinese research team also reported that the circRNA synthesis using Tetrahymena group I intron ribosome is not introduced with additional nucleotides.^98^

#### 2.3.2 Group II intron‐mediated circRNA synthesis

Other self‐cleaving ribozymes, including group II intron self‐splicing method, hairpin ribozymes (HRP) and hammerhead ribozymes, could also aid in generating circRNA.^99^, ^100^ Group II intron is derived from the yeast mitochondrial genome.^101^ Group II intron also utilizes a sophisticated self‐splicing mechanism characterized by two sequential transesterification reactions. Initially, a specific adenosine residue within the intron performs a nucleophilic attack on the 5′ splice site, resulting in the formation of a lariat intermediate with a unique 2′–5′ phosphodiester linkage. Subsequently, the 3′‐OH group of the upstream exon targets the 3′ splice site, facilitating exon ligation and the excision of the intron as a lariat structure. This lariat can then be processed further to generate a circRNA molecule (Figure 1C, right).^58^ Compared with the group I intron, the utilization of group II intron does not necessitate the presence of exon sequences to produce the desired circRNA sequence. As a result, it avoids the generation of circRNA with the scar sequence. Wang et al.^102^ published a novel in vitro RNA circularization technique based on group II intron circularization in 2022, suitable for large‐scale production. This technology enables efficient cotranscriptional circularization of RNA while avoiding nonspecific sequence insertions.^102^ Moreover, the resulting circRNA exhibits efficient translation in both cells and murine models without eliciting significant innate immune responses.^102^ Leveraging these advancements, they founded Circode Therapeutics. However, further research is still required to validate the wide applicability of this method. Several studies have revealed that linear RNA sequence containing HRP element could undergo self‐processing and circularization.^103^, ^104^, ^105^ However, these naturally occurring ribozymes are seldom utilized for large‐scale circRNA synthesis.^57^

#### 2.3.3 In vivo circRNA synthesis

In addition to in vitro circRNA synthesis, ribozymes have also been employed for in vivo circRNA synthesis. Professor Jaffrey's team has made significant contributions in this area. They initially observed that circRNA can be generated through metazoan tRNA splicing, and subsequently developed the “tricY” system, an in vivo circRNA expression system based on this biogenesis pathway.^106^ In 2019, they further optimized the system by substituting the tRNA endonuclease with the Twister ribozyme.^107^ This modification resulted in a significant increase in the expression level of circRNA products. The enhanced system was subsequently named Tornado (Twister‐optimized RNA for durable overexpression).^107^ In 2023, Professor Jaffrey's team provided additional evidence to support the superior performance of the Tornado system compared with previous methods for generating circRNA in cells.^108^ They showed that the Tornado system can effectively collaborate with virus‐like particles (VLPs) to ensure prolonged protein expression.^108^

## 3 OPTIMIZING circRNA FOR ENHANCED FUNCTIONALITY

The structure of circRNA typically comprises IRES, CDS, and facilitating loop sequences. Optimizations of these sequences and assembling the optimal elements together could significantly enhance protein expression, reduce its immunogenicity, and improve its stability, which eventually improve the performance of circRNA therapeutics.

### 3.1 Enhancing protein yield

**FIGURE 2:**
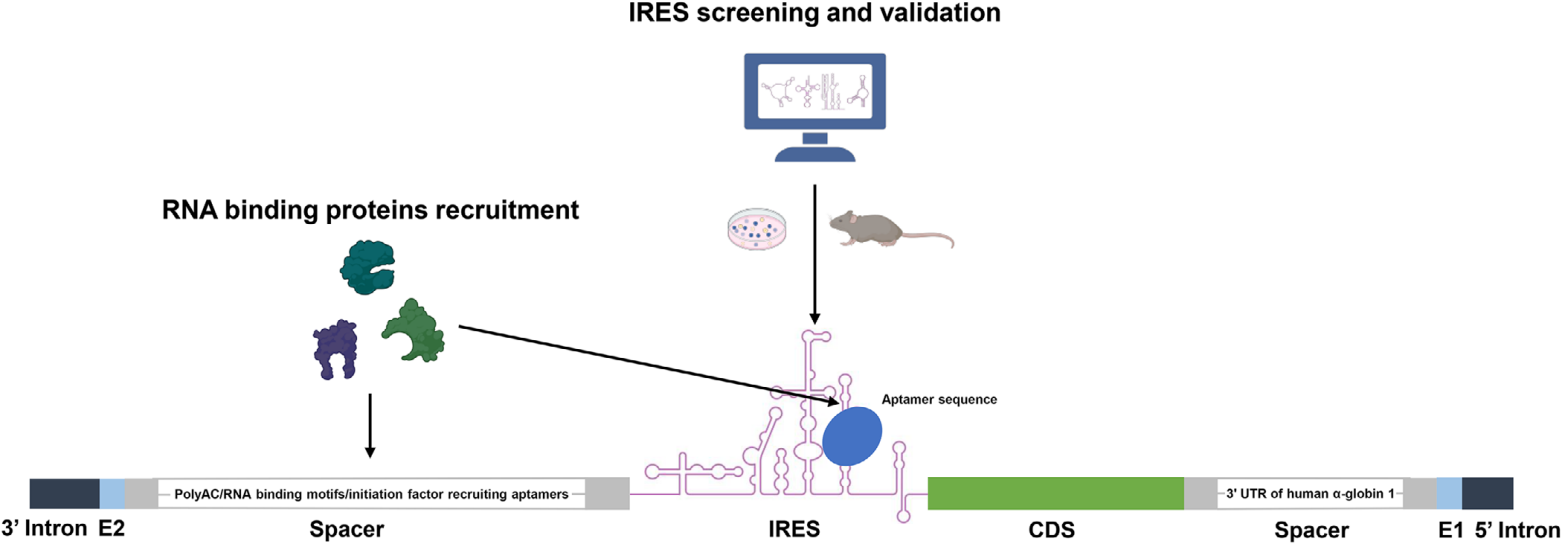
circRNA optimizations: protein yield enhancement. By screening and validating the most appropriate IRES elements, the protein expression capacity of circRNA can be significantly enhanced. Furthermore, the incorporation of elements such as PolyA/PolyAC, RNA binding motifs or initiation factor‐recruiting aptamers can also improve the translation efficiency of circRNA to a certain degree. circRNA, circular RNA; IRES, internal ribosome entry site.

At present, the efficacy of numerous circRNA‐based therapies hinges on the ability of circRNA to express therapeutic proteins within targeted cells, tissues, and organs. Consequently, augmenting the protein expression capabilities of circRNA is a pivotal factor in the advancement of circRNA therapeutics. Enhancement of circRNA protein expression can be primarily accomplished through IRES optimization, nucleic acid modifications, and refined spacer design (Figure 2).

#### 3.1.1 IRES screening and optimization

IRES, an essential element in cap‐independent translation, is located upstream of CDS and interacts with 40S ribosomal subunits to initiate translation.^109^ Currently, multiple IRES elements have been identified, which can be roughly classified into four types: type I, type II, type III, and type IV.^110^ Different types of IRES exhibit varying abilities in promoting protein translation, thus selecting the most suitable IRES element has become a significant challenge in the field of circRNA therapeutics.

In 2006, Mokrejs et al.^111^ established IRESite, a database compiling experimentally verified IRES structures. Accessible through the publicly available web interface http://www.iresite.org, this database serves as a valuable resource.^111^ Wesselhoeft et al.^43^ conducted a comparative analysis of commonly used IRES elements, identifying the coxsackievirus B3 (CVB3) IRES as the most efficient. It demonstrated approximately 1.5 times greater capacity to drive protein expression compared with the commonly used encephalomyocarditis virus IRES.^43^ Consequently, CVB3 IRES has been widely adopted in circRNA products.^112^, ^113^, ^114^ Chen et al.^115^ characterized various IRESs from different types and species, highlighting the potency of type I IRES in driving protein translation. Moreover, they identified several IRESs, including human rhinovirus (HRV)‐B3 and HRV‐B92, exhibiting superior translation capacity to CVB3 IRES.^115^ In the same year, the team developed a high‐throughput screening reporter assay to aid in the identification of superior IRES for circRNA.^42^ Utilizing this platform, the team screened and constructed a library of 40,855 oligonucleotide inserts (oligos) capable of driving circRNA expression and further identified 17,201 oligos that could drive circEGFP expression.^42^ The authors then compared these oligos with IRES reported in the IRESite database, finding a high match rate of 59.7%.^42^ This high‐throughput screening platform effectively identifies RNA sequences with IRES activity and functionality.^42^ To enhance circRNA protein expression, oRNA developed a high‐throughput screening platform named Formulated oRNA Cell‐based Evaluation.^116^ This platform supports the parallel arrayed synthesis, purification, LNP formulation, and cell‐based screening of IRES.^116^ Utilizing this system, nearly 3000 unique untranslated regions (UTRs) from viral genomes were screened, leading to the identification of hundreds of IRESs that facilitate translation in primary human T cells, hepatocytes, and myotubes.^116^ Certain IRESs from this screening enabled high levels of CAR expression in primary human T cells, which resulted in tumor regression in a human peripheral blood monocyte‐engrafted NALM6 tumor‐bearing mouse model.^116^ Our team demonstrated the efficient initiation of protein expression in circRNA using IRES elements from echoviruses (E), with E29 IRES performing comparably to CVB3 IRES.^114^ Our team expanded the investigations to screen IRES elements across different virus strains to identify the most suitable IRES for our Clean‐PIE circRNA.^68^ Using an algorithm‐assisted virtual high throughput screening method, we screened over 600 IRES elements.^68^ Subsequently, we verified their translational activity using firefly luciferase. Ultimately, we identified more than 20 IRES elements that demonstrated stronger capability to drive circRNA protein expression compared with the E29 IRES.

Artificial intelligence algorithms have also made significant contributions in predicting and optimizing IRES. Zhou et al.^117^ have developed a deep learning method for circRNA IRES prediction (DeepCIP). This method has demonstrated superior performance compared with other comparative methods, which enhanced the investigation of the coding potential of circRNA and contributed to the development of circRNA therapeutics.^117^ Xu et al.^118^, ^119^ have constructed a computational approach to achieve optimal design of the circRNA with improved translatability, stability, and circularization efficiency called circDesign based on the previously reported Linear Design system. By combining ORFs, IRES motifs and other functional regions, and then comparing various scoring metrics such as the overall minimum free energy, codon adaptation index, positional entropy and ensemble diversity using computer algorithms, the optimal combination was identified using circDesign.^118^ Subsequently, the application of the optimized circRNA in rabies virus and varicella‐zoster virus vaccines demonstrated significantly improved performance compared with the nonoptimized circRNA, as evidenced by both in vitro and in vivo experiments.^118^

The ability of a given IRES to facilitate protein translation in circRNA may vary across different cellular contexts. Therefore, the identification of IRES elements that exhibit optimal performance in target cells play a crucial role in enhancing the expression of the desired protein.

#### 3.1.2 Nucleotide modification of circRNA

Evidence suggests that 1‐methylpseudouridine (m1Ψ) modified mRNA exhibits reduced immunogenicity and enhanced protein expression capacity.^120^ It appears that circRNA could also reap benefits from m1Ψ modification. However, Wesselhoeft et al.^52^ reported that the complete substitution of uridine with m1Ψ could impede ribozyme activity, making it difficult for circularization. They further synthesized m1Ψ circRNA using the T4 RNA ligase method, but no protein expression was observed.^52^ Therefore, it seems that circRNA does not benefit from m1Ψ modification.

Studies have shown that m6A modification can facilitate the translation of endogenous circRNA by recruiting the initiation translation factor.^37^ However, it remains unclear whether synthetic circRNA can derive similar benefits from m6A modification. Wesselhoeft et al.^52^ have synthesized circRNA with 10% m6A modification, but the protein expression was found to be significantly lower compared with unmodified circRNA. However, Chen et al.^115^ demonstrated that circRNA with 5% m6A incorporation exhibited comparable protein expression to that of unmodified circRNA while reducing immunogenicity. Based on these findings, They recommend a 5% m6A modification for circRNA.^115^ Further and more extensive investigations are warranted to ascertain the potential benefits of m6A modification for synthetic circRNA.

#### 3.1.3 Spacer design and optimization

The polyA sequences in the 5′ and 3′ UTR of mRNA serves as a binding site for RNA‐binding proteins, facilitating translation enhancement.^121^ Wesselhoeft et al.^43^ have demonstrated that circRNA, incorporated with polyA sequences or polyAC spacers control to IRES, display enhanced protein expression capacity, potentially attributed to the increased unstructured separation between IRES and E1–E2. This greater separation may mitigate the potential for disruption of IRES folding and translation initiation.^43^ Chen et al.^115^ optimized the 5′ terminal of IRES by incorporating 50 nucleotides spacers encoding RNA‐binding motifs or initiation factor recruiting aptamer sequence, which significantly improve translation efficiency. The authors further replaced the 3′ spacer with different 3′ UTR sequences.^115^ Interestingly, they found that the majority of 3′ UTRs did not contribute significantly to enhanced translation in circRNA, except for the 3′ UTR of human α‐globin 1.^115^ Our research team has pinpointed the crucial role of spacer sequences between E1–E2 and CDS in circRNA protein expression.^68^ By integrating sections of polyAC and homology arm sequences into the spacer fragment, we delved into the effects of different polyAC lengths on protein expression capacity.^68^ Intriguingly, a 114‐nucleotide polyAC sequence emerged as optimal for enhancing protein expression.^68^ In addition, in another study, our team discovered that spacers between introns and IRES elements may prevent mutual interference, thereby aiding their folding and enhancing circRNA protein expression.^114^ Consequently, we redesigned the spacers upstream of the IRES to better separate the intron and IRES and used the RNAfold web tool to predict the folding performance of the IRES.^114^ We found that a new spacer, H1S1, significantly improved the protein translation efficiency of the E29 IRES.^114^ These results underscore the potential of polyA/polyAC sequence insertions in significantly boosting circRNA translational efficiency.

#### 3.1.4 Other

Chen et al.^115^ demonstrated a notable improvement in translation efficiency with the aptamer‐eIF4G inserted CVB3 IRES compared with the wild‐type counterpart. Moreover, they found that vector topology and spacer length influenced translational efficiency.^115^ Notably, positioning the IRES element directly upstream of the CDS enhanced translational efficacy.^115^ Additionally, the inclusion of 50‐nucleotide spacers in the scar sequence promoted circRNA translation.^115^

### 3.2 Mitigating immunogenicity

**FIGURE 3:**
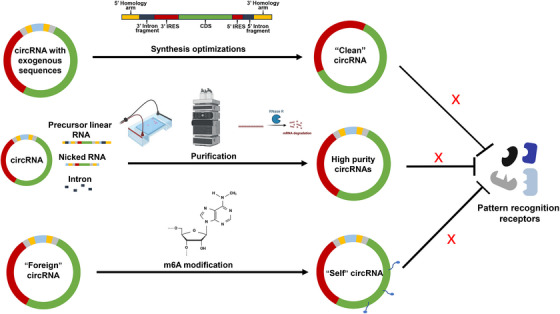
circRNA optimizations: immunogenicity mitigation. Top: by optimizing the synthesis process to minimize the introduction of exogenous sequences, inherent immune recognition of circRNA by the host can be reduced. Middle: techniques such as electrophoretic separations, HPLC and RNase R treatment can help diminish contamination from linear precursor RNA to some extent. Low: through m6A base modification, circRNA is perceived as “self,” thereby evading immune recognition. circRNA, circular RNA; HPLC, high performance chromatography; m6A, N^6^‐methyladenosine.

Mitigating the immunogenicity of exogenous RNA remains a significant challenge in circRNA therapeutics. Strategies to address this issue include minimizing by‐products generated during circRNA synthesis and reducing the incorporation of exogenous noncoding sequences (Figure 3).

#### 3.2.1 Optimizing synthetic methods for circRNA production

One of the reasons for increased immunogenicity of circRNA is the introduction of excess exogenous sequences during the synthesis process. Therefore, reducing the introduction of exogenous sequences becomes a key factor in lowering the immunogenicity of circRNA. Anabaena PIE and T4 td PIE method could introduce approximately 186 and 74 extra nucleotides into circle products, respectively.^52^, ^84^ This has been associated with high immunogenicity of circRNA produced by these methods.^122^ However, T4 RNA ligase methods only introduce two to three exogenous nucleotides during this process.^82^ In theory, circRNA generated using T4 RNA ligase methods has lower immunogenicity compared with those produced using the Anabaena PIE and T4 td PIE methods. Liu et al.^84^ have produced the same circRNA using these three methods and proved that Anabaena and T4 td circRNA induced high‐level expression of inflammatory cytokines including IFNβ, TNFα, IL‐6, and retinoic acid‐inducible gene‐I (RIG‐I). However, the cytokine levels induced by circRNA synthesized using T4 RNA ligase were significantly lower.^84^ Another method called the STS group I allows for the synthesis of RNA without introducing excess exogenous nucleotides.^97^ Lee et al.^97^ compared circRNA synthesized using the STS and PIE methods. Theoretically, the immunogenicity of circRNA without exogenous sequences should be significantly reduced. However, their data showed that after high performance chromatography (HPLC) purification, there was little difference in immunogenicity between the two methods.^97^ Our team has developed an optimized method called Clean‐PIE, which generate circRNA without extra nucleotides. circRNA produced by this method exhibited reduced immunogenicity compared with those produced by the Anabaena PIE method.^68^ For instance, inflammatory cytokines such as IFN‐β, IL‐6, and MDA5 were significantly lower in the circRNA produced by this method compared with those produced by the Anabaena PIE method.^68^ In conclusion, optimizing synthesis methods and procedures to reduce the introduction of exogenous sequences is one of the important strategies to minimize circRNA immunogenicity (Figure 3, top).

#### 3.2.2 Purification of circRNA products

As reported, contaminants arising from in vitro circRNA synthesis, including linear precursor RNA, nicked RNA, and intron fragments, can act as potent immunogens by engaging pattern recognition receptors, thereby eliciting robust innate immune responses.^122^, ^123^ Therefore, it is essential to devise effective purification protocols aimed at removing these impurities.

Electrophoretic separation, a fundamental laboratory‐scale purification technique, exploits the differential mobility of RNA species within an electric field. This method relies on distinct molecular weights and conformations to achieve precise separation of RNA molecules.^124^ Various gel types yield different band patterns and positions. On urea denaturing gel, circRNA migrates more slowly compared with its precursor RNA, whereas on native agarose gel, circRNA runs normally.^12^ Wesselhoeft et al.^43^, ^52^ employed E‐Gel EX electrophoresis (Thermo Fisher) to differentiate circRNA, nicked RNA, and precursor RNA. However, this method is deemed inefficient and unsuitable for large‐scale purification. RNase R digestion represents an efficient method for eliminating linear contaminants.^125^ The covalently closed single‐strand structure prevents circRNA from the degradation of this 3′–5′ exonuclease.^1^ However, the intricate secondary structure of linear RNA may impede RNase R digestion efficiency, potentially leading to the generation of truncated linear RNA.^125^ Chromatographic methods have been employed for the purification of oligonucleotides since 1970s.^126^ HPLC, such as size‐exclusion chromatography, is frequently utilized in RNA purification.^127^ Size‐exclusion chromatography is adept at separating RNA molecules with notable length discrepancies, such as introns produced during synthesis. However, for molecules of similar size, such as circRNA, linear precursor RNA, and nicked RNA, the separation efficiency is comparatively diminished^128^ (Figure 3, middle).

In summary, the thorough elimination of linear RNA impurities is essential to mitigate potential immune responses. Electrophoresis or HPLC methods offer effective means to obtain highly pure circRNA. Sole reliance on RNase R may pose challenges in achieving high circRNA purity. Of these approaches, HPLC presents greater scalability, rendering it preferable for large‐scale circRNA purification. Moreover, the synergistic application of multiple purification methods can augment circRNA purity further.

#### 3.2.3 m6A modification of circRNA

Previous studies have indicated that m6A modification of 5′‐P linear RNA can impede the binding and activation of RIG‐I.^129^ However, whether circRNA can attenuate its immunogenicity through m6A modification remains uncertain. Chen et al.^123^ proposed that m6A modification labels circRNA as “self,” thereby mitigating its immune response. They compared the levels of inflammatory cytokines after transfecting cells with linear RNA, unmodified circRNA, 1% m6A‐modified circRNA, and 100% m6A‐modified circRNA.^123^ Their findings showed that 1% m6A modification significantly reduced the immunogenicity of circRNA, while 100% m6A‐modified circRNA exhibited extremely low immunogenicity, comparable to that of linear RNA.^123^ Conversely, Wesselhoeft et al. observed that m6A modification of circRNA is disruptive and can interfere with the circularization process, contrasting with Chen et al.’s findings.^52^, ^115^, ^123^ Currently, consensus on the effects of m6A modification on circRNA is lacking, warranting further exploration (Figure 3, low).

### 3.3 Enhancing circularization efficiency

The substantial decline in circRNA circularization efficiency is predominantly ascribed to lengthier sequences, particularly the extended intervening regions between splice sites. These prolonged regions may attenuate the interaction capability between splice sites, impeding the formation of a stable complex and consequently diminishing splicing efficiency. Nevertheless, therapeutic applications of circRNA typically demand sequences spanning several thousand nucleotides. Thus, there is an imperative to explore methodologies aimed at enhancing circularization efficiency.

**FIGURE 4:**
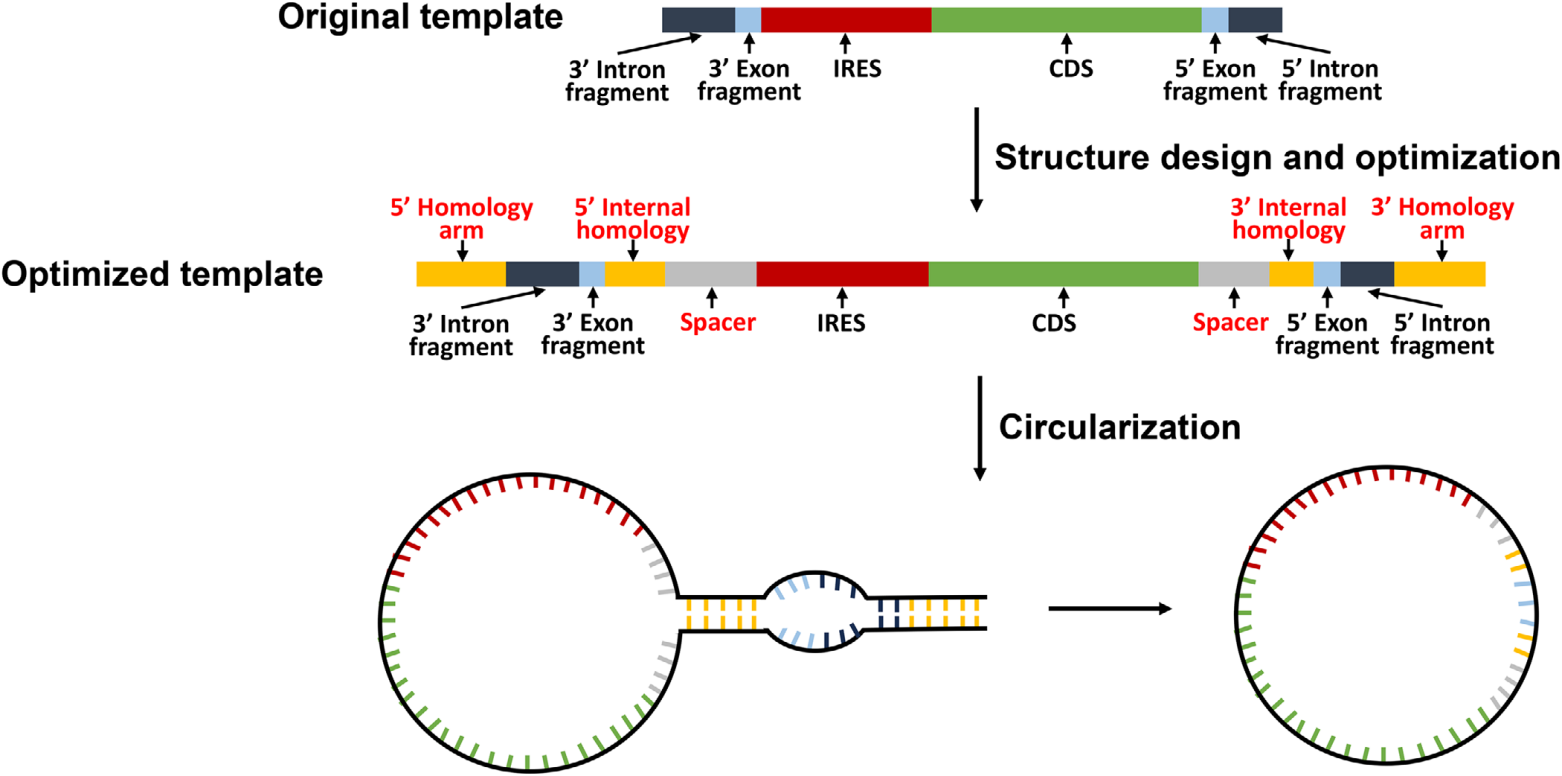
circRNA optimizations: circularization efficiency improvements. The integration of complementary homologous arms and spacers into the primary backbone markedly enhances the circularization efficiency. circRNA, circular RNA.

Wesselhoeft et al.^43^ devised an ingenious approach by strategically engineering perfectly complementary homolog arms positioned at the 5′ and 3′ termini of precursor linear RNA. This modification facilitates the interaction between the 5′ splice site and 3′ splice site, thereby promoting circRNA formation with heightened efficiency, reaching up to 48%^43^ (Figure 4). To further refine their method, they introduced spacers between the 3' PIE splice site and IRES to mitigate interference from the IRES on splicing ribozyme activity.^43^ Significantly, their results demonstrated that the inclusion of spacers substantially enhanced circularization efficiency, elevating it from 46% to an impressive 87%.^43^ These optimized techniques were subsequently applied to two canonical PIE systems, Anabaena and T4 td. Interestingly, the engineered Anabaena PIE system outperformed the T4 td PIE system, showing a 95% increase in splicing efficiency and approximately 37% reduction in circRNA nicking.^43^ In subsequent optimized circRNA circularization strategies based on the oRNA PIE backbone, the inclusion of homology arms to enhance circularization efficiency has been retained.^68^

Essentially, improving circRNA circularization efficiency hinges on incorporating elements that facilitate interaction between the 5′ and 3′ splicing sites, while also minimizing mutual interference between critical circularization components. Significantly enhancing the circularization efficiency of circRNA not only reduces costs but also substantially decreases the production of byproducts. This stands as a pivotal focal point for advancements in circRNA therapeutics.

### 3.4 Enhancing stability

The elevated stability of circRNA in comparison with linear RNA presents a notable advantage.^43^ However, it is evident that circRNA still exhibits a degree of fragility relative to other small molecule counterparts. Therefore, it is imperative for circRNA therapeutics to investigate further strategies aimed at enhancing circRNA stability

Liu et al.^84^ compared the stability of circRNA synthesized via the RNA ligase I method with those synthesized using PIE methods. After transfecting equal amounts of circRNA into two different cell lines, RNA was extracted at various time points and analyzed via gel electrophoresis.^84^ The results showed that circRNA synthesized via the RNA ligase I method did not exhibit a decline at 48 h, whereas those synthesized using PIE methods showed a significant decrease by 24 h.^84^ qPCR results further confirmed these findings.^84^ Furthermore, Chen et al.^115^ investigated the stability of 5% m6A‐modified circRNA in fetal bovine serum (FBS) solutions with varying concentrations, suggesting that m6A modification may enhance stability. Their results indicated that 5% m6A‐modified circRNA only fully degraded in a 1.5% FBS solution, whereas unmodified circRNA completely degraded in a 1% FBS solution.^115^ Therefore, they concluded that m6A modification could enhance the stability of circRNA. However, another study reported that m6A‐modified circRNA could be degraded via an endoribonucleolytic cleavage pathway (YTHDF2–HRSP12–RNase P/MPR pathway).^130^ The potential for m6A modification to enhance the stability of circRNA warrants further investigation. Xu et al.^118^ have developed circDesign, an algorithmic platform for predicting circRNA structures and designing sequences. circDesign employs Minimum Free Energy and Codon Adaptation Index to screen ORF region sequences, considering the stability and translational efficiency of ORFs. These sequences are then integrated with functional elements, including IRES and exon segments, to assemble complete circRNA molecules.^118^ The Minimum Free Energy algorithm posits that RNA secondary structures become more stable as free energy decreases.

In summary, optimizing circRNA synthesis strategies, implementing specific nucleic acid modifications, and employing artificial intelligence‐assisted circRNA structural design may significantly enhance the stability of circRNA. However, given the inherent stability advantage of circRNA over linear RNA, research efforts aimed at enhancing circRNA stability have been relatively limited. Further investigation is warranted to bolster circRNA in this aspect.

## 4 PLATFORM FOR circRNA DELIVERY

Generally, RNA molecules struggle with efficient cellular entry due to their hydrophilic nature and negative charge.^47^ Unprotected RNA is susceptible to rapid degradation by nucleases in bodily fluids and is swiftly cleared from the host via renal filtration, leading to a half‐life of less than 7 min.^131^ Even if a small quantity of RNA successfully enters cells, it may be degraded by enzymes during endosomal transport and within acidic lysosomes.^131^ Thus, an effective delivery system is essential for circRNA therapeutics.

Currently, delivery vectors can be broadly categorized into viral and nonviral vectors.^47^, ^132^, ^133^, ^134^ Viral vectors are the most commonly used systems in gene therapy, primarily including adenoviral vectors, retroviral vectors, and adeno‐associated viral (AAV) vectors, with AAV vectors being the most widely used.^135^, ^136^ Nonviral vectors utilize physical methods, chemical methods, and biological‐derived vectors to transfect therapeutic genetic material.^47^, ^137^, ^138^ Physical methods include gene guns,^139^, ^140^ electroporation^141^, ^142^, ^143^ and sonoporation,^144^, ^145^ among others. Chemical methods include LNP,^146^, ^147^, ^148^, ^149^, ^150^ polymers,^147^, ^151^, ^152^ VLP,^153^, ^154^, ^155^, ^156^ biomimetic and inorganic nanoparticles,^157^, ^158^ among others. Biological‐derived vectors include extracellular vesicle (EV),^159^, ^160^ bacteria membrane vesicle^161^, ^162^ and red blood cell membrane,^163^, ^164^ among others. Currently, nonviral vectors are the mainstream carriers for therapeutic delivery of circRNA (Table 2).

**TABLE 2:** Delivery vectors for circRNA therapeutics.

Delivery vectors	Viral vector^66^, ^137^	Electroporation^165^, ^166^	LNP^66^, ^137^, ^138^	Exosome^66^
Categorization	AAV, adenoviral, retroviral vector, etc.	Bulk electroporation, in‐flow electroporation, nano‐electroporation, etc.	Solid LNPs, polymers, etc.	–
Vector size	120–150 nm	–	10–1000 nm	40–160 nm
Cargo types	ssDNA, dsDNA, etc.	circRNA, oligonucleotides, pDNA, mRNA, etc.	circRNA, oligonucleotides, pDNA, mRNA, etc.	circRNA, oligonucleotides, pDNA, mRNA, etc.
Cargo size	5–15 kb	There is no length restriction, provided they can pass through the membrane pores	There is no length restriction, provided they can be encapsulated by LNP	There is no length restriction, provided they can be encapsulated by exosome
Scalability	High	Moderate	High	Low
Cell uptake mechanism	Receptor‐mediated endocytosis, direct fusion, direct injection, etc.	Electro‐membrane permeabilization	Receptor‐mediated endocytosis, membrane fusion, etc.	Receptor‐mediated endocytosis, membrane fusion, direct membrane penetration, etc.
Transfection efficacy	Variable	Medium	Low/medium	Need more explorations
Immunogenicity	High	Low	Medium/high	Low
Advantages	Relatively mature manufacturing, stable gene expression	Refined procedures, uncomplicated process	Tailored platform, cost effective, high‐capacity, and easily scalable	Good biocompatibility, low immunogenicity, natural carrier, and ability to cross biological barriers
Disadvantages	Complex production process, expensive, limited payload capacity, high immunogenicity, prolonged safety concerns, etc.	Major biological disruptions, including reduced cell viability, cell damage, phenotype modifications, and alterations in gene expression, etc.	Suboptimal transfection efficiency for several primary cells, potential for off‐target gene transfer, requirement for stringent manufacturing standards, materials‐related toxicity, etc.	Low yield, complex isolation and purification, scalability issue, etc.

Abbreviations: LNP, lipid‐based nanoparticle; ssDNA, single‐strand DNA; dsDNA, double‐strand DNA; pDNA, plasmid DNA; mRNA, messenger RNA; circRNA, circular RNA; kb, kilobase.

### 4.1 Viral vector

Viral vectors are seldom employed for the delivery of circRNA. When viral delivery is required, DNA must serve as an intermediate. The viral vectors first integrate the DNA into the host genome, followed by its transcription into RNA, which subsequently undergoes circularization within the cell to form circRNA.^133^

Meganck and colleagues^167^ engineered recombinant AAV vectors that incorporate transgene cassettes containing intronic sequences designed to facilitate backsplicing, resulting in the production of circRNA transcripts. By leveraging this vector platform, they successfully delivered large quantities of circRNAs with transgene expression to specific organs, such as the heart and brain.^167^ Their research underscores the potential of AAV‐based circRNA expression systems for studying circRNA function and tissue‐specific regulation in animal models.^167^ Three years later, the same authors reported a study using AAV‐mediated circRNA to explore elements essential for circRNA formation and leverage these findings to enhance the design and expression of synthetic circRNAs.^168^ Lavenniah et al.^169^ utilized AAV to deliver circRNA‐based microRNA (miR)‐132 and miR‐212 sponges to the cardiomyocytes of a mouse model with cardiac disease. These circular sponges could competitively inhibit miR‐132 and miR‐212 activity, thereby restoring the cardiac function in treated mice.^169^ Similarly, Lu et al.^41^ employed AAV to deliver a circRNA‐based protein sponge, called circRNA insulin receptor (circ‐INSR), to the cardiomyocytes of a mouse model with chronic doxorubicin‐induced cardiotoxicity. The circ‐INSR was capable of interacting with the single‐stranded DNA‐binding protein and mediate cardioprotective effects under doxorubicin stress.^41^ In 2022, two research teams independently published similar studies where they developed circular adenosine deaminase acting on RNA (ADAR)‐recruiting guide RNAs.^170^, ^171^ By delivering these guide RNAs into various cell lines using AAV, they achieved robust and durable RNA editing effects. Ultimately, they successfully corrected numerous nonsense mutations in a mouse model of Hurler syndrome using this approach.^170^, ^171^

Due to the inherent risk of gene toxicity from host genome insertion with virus‐mediated in vivo circRNA delivery, this method does not offer significant advantages over other delivery methods. However, it is highly beneficial for exploring the functions and regulatory mechanisms of circRNA within cells exhibiting viral tropism.

### 4.2 Electroporation

Electroporation is a highly versatile and efficient nonviral method for RNA delivery that uses brief, high‐voltage electrical pulses to create temporary pores in cell membranes, enabling the entry of RNA molecules while being suitable for a wide range of cell types, including those difficult to transfect.^165^, ^166^, ^172^ Electroporation is increasingly utilized in mRNA therapy due to its efficiency and versatility.^173^, ^174^, ^175^, ^176^ However, its application in circRNA delivery remains relatively limited.

Fan et al.^177^ delivered circRNA mSCAR into macrophage mitochondria using exosome and electroporation‐based systems can reduce systemic inflammation and mortality in sepsis by promoting M2 polarization, highlighting circRNA mSCAR's therapeutic potential. Liu et al.^178^ have developed a safe and effective nanochannel electro‐injection (NEI) system for delivering various nucleic acid molecules into dendritic cells (DCs). Their results show that this system can transfect DC2.4 cells with fluorescent dye, plasmid DNA, mRNA, and circRNA with efficiencies exceeding 70% and maintaining good biosafety (viability > 85%).^178^ Additionally, the NEI system achieved a 68.3% transfection efficiency of circRNA into primary mouse bone marrow DCs without significantly affecting cell viability or inducing DC maturation.^178^ These findings suggest that NEI could serve as a safe and effective in vitro transfection platform for DCs, with promising potential for developing anticancer DC vaccines. Our team has developed a TCR‐T therapy targeting human cytomegalovirus (CMV) pp65 through electroporation of circRNA.^111^ The efficacy of this therapy has been validated in both in vitro and in vivo experiments. This approach offers a safe and effective treatment option for controlling CMV infections, especially following allogenic hematopoietic stem cell transplantation. In addition, our team is dedicated to researching electroporation of circRNA for CAR‐T cell production. Currently, we have successfully constructed CAR‐T cells targeting Delta‐like ligand 3 (DLL3) for small cell lung cancer using a circRNA‐electroporation strategy. These CAR‐T cells have demonstrated promising tumor‐killing effects both in vitro and in vivo (data not published). Overall, compared with other delivery methods, the advantage of electroporation is its ability to transfect hard‐to‐transfect cells effectively. However, a limitation of this method is that the process can cause cellular damage, leading to reduced cell viability posttransfection.

### 4.3 Lipid nanoparticle

LNPs are currently the most widely used RNA delivery vectors, having been approved by the U.S. Food and Drug Administration (US FDA) for the delivery of two mRNA‐based COVID‐19 vaccines^45^, ^46^, ^179^ and for an siRNA therapy to treat hereditary transthyretin‐mediated amyloidosis.^180^ Many ongoing preclinical and clinical studies are employing LNPs as delivery systems for circRNA. An LNP is typically composed of four main components: ionizable lipids, cholesterol, phospholipids, and a polyethylene glycol (PEG) lipid, each serving distinct functions.^149^ Ionizable lipids, making up roughly 20%‐40% of the total lipids, are the key component of LNPs and crucial for delivery efficiency.^150^ Various ionizable lipids, such as ALC‐0315, SM‐102, and MC3 have been approved for RNA delivery due to their unique physicochemical properties.^181^, ^182^ Cholesterol, a natural component of cell membranes, is primarily found in the LNP shell, enhancing stability and aiding endosomal escape.^149^ Screening of various cholesterol analogues has identified those that improve endosomal escape and cargo release efficiency.^183^, ^184^ Phospholipids, known as helper lipids, self‐assemble into lipid bilayers and play a significant role in encapsulating nucleic acids, forming LNPs, and facilitating endosomal escape.^149^, ^150^ 1,2‐Distearoyl‐sn‐glycero‐3‐phosphocholine and 1,2‐dioleoyl‐sn‐glycero‐3‐phosphoethanolamine (DOPE) are commonly used helper lipids.^185^ The PEG lipid forms a polymerization layer on the LNP shell, preventing serum protein absorption and mononuclear phagocyte system uptake, thus extending circulation half‐life and preventing LNP aggregation during storage and transportation.^186^ However, PEG lipids may hinder the interaction between nanoparticles and target cells, reducing transfection efficiency.^187^ Continuous research aims to optimize the proportion, length, and molecular weight of PEG lipids for better efficacy and safety.^188^, ^189^

Various methods have been employed in the manufacture of LNPs, including microfluidic method,^190^ reverse‐phase evaporation method,^191^ thin film hydration method,^192^ and solvent injection method.^193^ Among these techniques, microfluidic methods are widely favored for LNP production. This approach involves dissolving phospholipids, ionizable lipids, cholesterol, and PEG lipids in an ethanol phase at specific molar ratios, while RNA is dissolved in an acidic aqueous phase.^149^, ^194^ Utilizing a microfluidic chip within the device, which contains distinct channels for the ethanol and aqueous phases, facilitates the precise mixing of these components and the subsequent self‐assembly of LNPs. During this assembly process, targeted ligands such as antibodies, peptides, aptamers, and other motifs can be introduced to the LNP surface to enhance delivery efficiency.^149^, ^150^, ^152^, ^195^, ^196^

Qu et al.^197^ utilized commercial LNPs (Precision NanoSystems) to encapsulate circRNA encoding the SARS‐CoV‐2 receptor‐binding domain (RBD) protein antigen. Following intramuscular injection in mice and rhesus monkeys, the results demonstrated that this circRNA vaccine effectively elicited neutralizing antibodies, providing protection against SARS‐CoV‐2 and its variants.^197^ Li et al.^198^ developed a microfluidics‐based platform for synthesizing LNPs using multi‐armed ionizable lipids. They systematically compared their LNPs to the US FDA‐approved LNPs, MC‐3, and SM‐102. Following intramuscular injection of LNPs encapsulating circRNA encoding the Ovalbumin (OVA) antigen in mice, peripheral blood was collected at 24 h for proinflammatory cytokine analysis.^198^ The results showed that their LNPs elicited higher cytokine levels without significant damage to major organs.^198^ Zhou et al.^199^ employed LNPs formulated with the same composition as the BNT162b2 mRNA vaccine^46^ to encapsulate circRNA encoding the monkeypox virus (MPV) surface antigen proteins, effectively safeguarding mice against monkeypox infection. Xu et al.^200^ utilized a high‐throughput combinatorial approach for synthesizing and screening LNPs optimized for efficient delivery of circRNA to lung tumors. Ultimately, they identified the top‐performing LNP, designated as H1L1A1B3.^200^ Compared with the traditional ALC‐0315 LNP, H1L1A1B3 demonstrated a fourfold increase in circRNA transfection efficiency in lung cancer cells. A single intratumoral injection of H1L1A1B3 LNPs loaded with circRNA encoding IL‐12 elicited a robust immune response in a Lewis lung carcinoma model, resulting in significant tumor regression.^200^ oRNA Therapeutics developed immunotropic LNPs that exhibit preferential biodistribution to the spleen. These LNPs encapsulate circRNA encoding an anti‐CD19 CAR construct, and upon intravenous injection into mice, they enable in situ generation of CAR‐T cells, effectively reducing tumor burden in the treated mice.^116^ Recently, our team synthesized a biodegradable and ionizable glycerolipid, TG6A, through a three‐step esterification process.^201^ The molecular structure of TG6A includes a tertiary amine head group, a glycerol linker, and branched alkane tails. TG6A‐LNPs were formulated using TG6A, DOPE, cholesterol, and DMG‐PEG in a molar ratio of 50:10:38.5:0.75.^201^ circRNA encoding the target protein was encapsulated via electrostatic interactions between the ionizable amine groups of TG6A and the phosphate groups of the circRNA molecules.^201^ The TG6A‐LNPs we prepared achieved a transfection efficiency of 90% for EGFP in mesenchymal stem cells.^201^ Using this LNP‐circRNA strategy, mesenchymal stem cells effectively expressed the fibroblast growth factor (FGF)18 protein, which enhances cartilage regeneration, significantly promoting cartilage repair in a mouse model.^201^ Additionally, our team is developing an in vivo CAR‐T therapy based on LNP‐circRNA technology. We use CD5 antibody‐modified LNPs to encapsulate circRNA encoding the CAR structure and inject them intravenously into mice. Analysis of CD3‐positive T cells from the spleen shows a CAR expression rate of approximately 15–20% (data not published).

Despite the widespread application of LNPs in small molecule drug delivery, they still exhibit certain limitations such as instability during storage, potential toxicity from lipid components, variability in encapsulation efficiency, and challenges in achieving targeted delivery to specific tissues or cells.^202^ Addressing these issues is crucial for expanding the application of LNPs.

### 4.4 Exosome

Exosomes are small lipid bilayer vesicles, typically 40–160 nm in diameter, that facilitate the transfer of biomolecules such as proteins, lipids, and nucleic acids between cells, crucial for enabling intercellular communication.^203^, ^204^ Its architecture includes a natural lipid bilayer enriched with adhesion proteins, making them less toxic and better tolerated in the body compared with synthetic nanoparticles.^205^ This design also helps evade sequestration by mononuclear phagocytes, improving drug delivery to target cells and significantly boosting therapeutic efficacy.^205^, ^206^ Therefore, exosomes represent a natural and highly promising vehicle for in vivo drug delivery. Various methods can be used to extract extracellular exosomes, including sucrose‐gradient ultracentrifugation and commercial extraction kits, among others. Currently, there are studies that load RNAs such as siRNA, mRNA, and circRNA into exosomes for gene therapy. The methods for RNA loading can be divided into exogenous and endogenous loading.^207^ Exogenous loading involves isolating exosomes from target cells and then incubating them with the cargo to achieve passive loading or using physical methods such as electroporation for active loading. However, electroporation can alter the morphology and physiological structure of exosomes, potentially reducing delivery efficiency.^208^ Endogenous loading involves transfecting parent cells with the intended cargo, leading to the production of large quantities of the target RNA within the cells. This RNA is then subsequently packaged into exosomes and secreted extracellularly.^207^

Many studies are currently exploring the use of exosomes as delivery vehicles. Fan et al.^177^ developed an exosome‐based nanoplatform, ExoMito, for mitochondrial delivery of circRNA mSCAR, addressing its decrease in septic mice macrophages associated with excessive M1 polarization. This system, using electroporated exosomes with poly‐d‐lysine‐graft‐triphenylphosphine, successfully delivered circRNA mSCAR into mitochondria, promoting M2 macrophage polarization, reducing systemic inflammation, and improving survival rates in septic models.^177^ Yang et al.^209^ used rabies virus glycoprotein (RVG) engineered extracellular vesicles to deliver circSCMH1 to the brain, improving functional recovery after ischemic stroke in rodent and nonhuman primate models by enhancing neuronal plasticity, inhibiting glial activation, and reducing immune cell infiltration. The team also used RVG‐extracellular vesicles to deliver circDYM directly to the brain, demonstrating significant alleviation of depressive‐like behaviors in a chronic unpredictable stress mouse model.^210^ Their study highlights the potential of exosome‐based delivery systems in targeting and treating major depressive disorder by overcoming blood–brain barrier limitations and reducing neuroinflammation.^210^

Despite the significant potential of exosomes for drug delivery, several challenges remain. First, exosomes derived from different cell types can exhibit heterogeneity in their contents and functions, complicating quality control efforts.^211^ Second, the purification and modification of exosomes are still major challenges.^212^, ^213^ Additionally, exosome storage requires stringent conditions, posing difficulties for transport and storage.^214^ Last, current extraction techniques are limited, which constrains the ability to scale up production.^215^ Overcoming these challenges is essential for the clinical translation of extracellular vesicles as drug delivery vehicles in the future.

## 5 circRNA‐BASED THERAPEUTICS

circRNA has emerged as a hotspot in novel disease therapy research. circRNA can be categorized into endogenous and synthetic types, each playing distinct roles in disease treatments. Endogenous circRNA, due to their functions as RNA sponges and protein sponges, are closely associated with the therapeutic efficacy and progression of various diseases, making them potential targets for treatment. Currently, numerous reviews have thoroughly detailed the advancements of endogenous circRNA in disease initiation, progression, metastasis, and therapeutic efficacy.^1^, ^216^, ^217^ In recent studies, Li et al.^218^ reported that circPDIA3 induces oxaliplatin resistance in colorectal cancer by inhibiting pyroptosis through blocking GSDME‐C domain palmitoylation, with a positive feedback loop involving circPDIA3/miR‐449a/XBP1 enhancing chemoresistance. Ji and colleagues^219^ identified a circRNA called Hsa‐circ‐0003764 (circPTPN12) from the circRNA database, discovering its role in suppressing hepatocellular carcinoma progression by interacting with the PDZ domain of PDLIM2, promoting P65 ubiquitination and the assembly of the PDLIM2/OTUD6B complex, thereby highlighting the ESRP1/circPTPN12/PDLIM2/NF‐κB axis as a potential therapeutic target. Song et al.^220^ demonstrated that circCAPG could enhance triple‐negative breast cancer cell proliferation and metastasis by encoding the polypeptide CAPG‐171aa, which activates the MEKK2–MEK1/2–ERK1/2 pathway, and is regulated by SLU7, suggesting it as a potential prognostic biomarker and therapeutic target. It is anticipated that more endogenous circRNAs will be identified in the future, with their roles in disease regulation being comprehensively analyzed, providing powerful targets for disease‐specific therapies.

**FIGURE 5:**
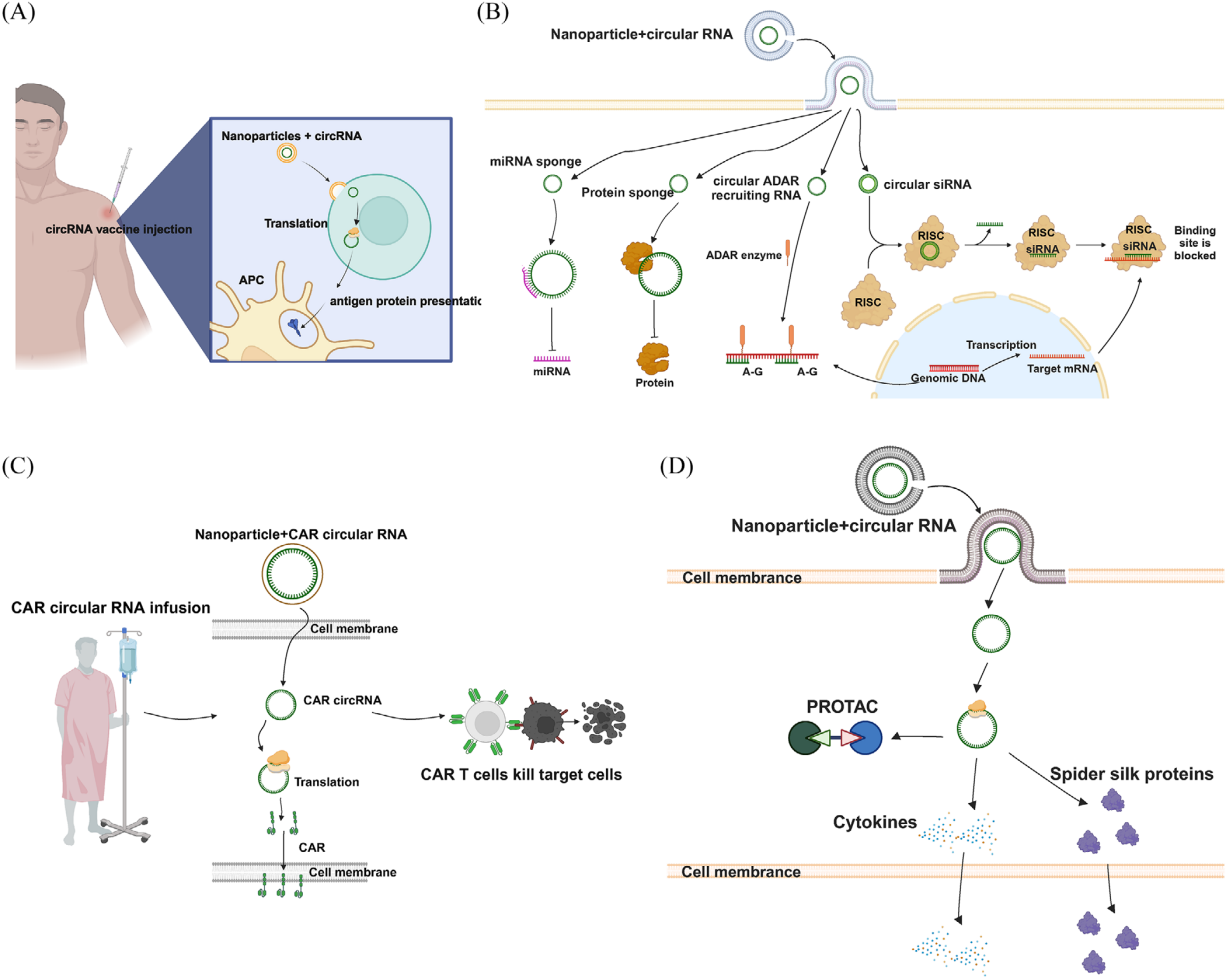
The applications of circRNA therapeutics. (A) Vaccine. circRNA encoding antigenic protein are encapsulated by nanoparticles and injected into the human body. Subsequently, the circRNA enter cells and express the corresponding antigenic protein, which are ultimately presented to APCs, eliciting an immune response in the body; (B) gene expression modulation. By synthesizing circRNAs with functions such as miRNA sponge, protein sponge, gene silencing, and so on and then utilizing nanoparticles to facilitate their entry into target cells, these circRNAs can exert their respective gene regulatory functions; (C) adopt cell therapy. By encapsulating circRNAs encoding CAR/TCR structural proteins in nanoparticles with targeting capabilities, CAR‐T/TCR‐T cells can be directly generated in the human body through intravenous injection, eliminating the need for laborious ex vivo viral modification processes; (D) other. Utilizing the protein translation capability of circRNA, theoretically, we can express desired proteins such as cytokines regulating the tumor immune microenvironment, spider silk proteins, and PROTAC complexes that degrade specific proteins (created with BioRender.com). circRNA, circular RNA; APC, antigen‐presenting cell; CAR, chimeric antigen receptor; TCR, T cell receptor; PROTAC, proteolysis‐targeting chimeras. ADAR, adenosine deaminase acting on the RNA; RISC, RNA‐induced silencing complex.

In recent years, advances in RNA synthesis technologies^43^, ^68^ and the recognition of the advantages of circRNA in expressing target proteins with high stability^56^, ^221^ have led to the emergence of therapeutic approaches utilizing synthetic circRNA to express specific proteins with therapeutic potential. Among the most prominent areas are vaccines, gene expression regulation, adoptive cell therapies, and protein replacement therapies (Figure 5 and Table 3).

**TABLE 3:** Comprehensive overview of published circRNA therapeutics.

Study	Categorization	Synthetic method	Delivery vector	Product	Disease	Stage
2022 Qu et al.^197^	Vaccine	Ribozyme ligation	LNP	Spike protein termed RBD	COVID‐19	Preclinical
2022 Seephetdee et al.^222^	Vaccine	Ribozyme ligation	LNP	Spike trimer termed VFLIP	COVID‐19	Preclinical
2022 Li et al.^198^	Vaccine	Ribozyme ligation	LNP	OVA	Colorectal cancer, melanoma	Preclinical
2024 Wan et al.^223^	Vaccine	Ribozyme ligation	LNP	RVG	Rabies virus	Preclinical
2024 Zhou et al^199^	Vaccine	Ribozyme ligation	LNP	Different surface protein antigens of MPV including A29L, A35R, B6R, and M1R	MPV	Preclinical
2022 Mabry et al.^116^	CAR‐T	Ribozyme ligation	LNP	Anti‐human CD19 CAR	Acute human B lympholeukemia	Preclinical
2023 Shen et al.^111^	TCR‐T	Ribozyme ligation	Electroporation	Anti‐pp65 TCR	CMV	Preclinical
2024 Wang et al.^224^	Vaccine+CAR‐T	Ribozyme ligation	LNP	HER2 fusion protein, anti‐HER2‐CAR	Colorectal cancer, breast cancer	Preclinical
2022 Yang et al.^114^	Cytokine	Ribozyme ligation	LNP	IL‐15, IL‐12 single chain, granulocyte macrophage colony‐stimulating factor, and IFN‐α 2b	Lung cancer, colorectal cancer, melanoma	Preclinical
2024 Xu et al.^200^	Cytokine	Ribozyme ligation	LNP	IL‐12	Lung cancer	Preclinical
2024 Wang et al.^225^	Cytokine	Ribozyme ligation	LNP	IL‐12	Solid tumor	Preclinical
2024 Huang et al.^201^	Protein replacement therapy	Ribozyme ligation	LNP	FGF 18	Osteoarthritis	Preclinical
2022 Yang et al.^226^	Other	Ribozyme ligation	Intratumor injection	Anti‐PCNA PROTAC	Melanoma	Preclinical
2018 Zhang et al.^227^	Gene expression modulation	Chemical synthesis	Intratumor injection	GFP/luciferase‐siRNA	–	–
2018 Liu et al.^228^	Gene expression modulation	T4 enzymatic ligation	Lipofectamine RNAiMax	miR‐21 sponge	Gastric cancer	Preclinical
2019 Wang et al.^229^	Gene expression modulation	T4 enzymatic ligation	Lipofectamine RNAiMax	miR‐21 and miR‐93 sponges	Esophageal carcinoma	Preclinical
2020 Lavenniah et al.^169^	Gene expression modulation	Ribozyme ligation	AAV	miR‐132 and miR‐212 sponges	Cardiac hypertrophy	Preclinical
2022 Lu et al.^41^	Gene expression modulation	T4 enzymatic ligation	AAV	circRNA insulin receptor	Doxorubicin‐induced cardiac dysfunction	Preclinical
2020 Schreiner et al.^230^	Gene expression modulation	Ribozyme ligation	Lipofectamine MessengerMax	Heterogeneous nuclear ribonucleoprotein L sponge	–	–
2022 Yi et al^170^	Gene expression modulation	Ribozyme ligation	AAV	ADAR‐recruiting RNA	Hurler syndrome	Preclinical
2022 Katrekar et al.^171^	Gene expression modulation	Ribozyme ligation	AAV	ADAR‐recruiting RNA	Hurler syndrome	Preclinical
2024 Guo et al.^231^	Gene expression modulation	T4 enzymatic ligation	LNP	circRNA aptamer	Psoriasis	Preclinical

Abbreviations: RBD, the receptor‐binding domain; VFLIP, five (v) prolines, flexibly‐linked, inter‐protomer disulfide; OVA, ovalbumin; RVG, rabies virus glycoprotein; CMV, cytomegalovirus; MPV, monkeypox virus; FGF, fibroblast growth factor; PCNA, proliferating cell nuclear antigen; PROTAC, proteolysis targeting chimera; AAV, adeno‐associated virus; ADAR, adenosine deaminase acting on RNA; miR, microRNA; CAR‐T, chimeric antigen receptor T‐cell therapy; TCR‐T, T‐cell receptor engineered T cell therapy.

### 5.1 Vaccine

In the realm of vaccines, two prominent categories stand out: inactivated protein vaccines and mRNA vaccines. The remarkable success of mRNA vaccines in combatting the COVID‐19 pandemic has paved the way for research on circRNA vaccine.^45^, ^46^

In 2022, Professor Wensheng Wei and colleagues developed circRNA vaccines against SARS‐CoV‐2 and emerging variants.^197^ Their findings revealed that circRNA vaccines can achieve sustained and abundant expression of antigen proteins, resulting in higher levels of neutralizing antibodies in mouse and rhesus macaques compared with mRNA vaccines.^197^ This research holds historical significance in advancing circRNA therapeutics. In response to COVID‐19 variants, Seephetdee^222^ have also developed a circRNA‐based vaccine based on a new version of the spike trimer, termed VFLIP, which induces robust and balanced immune responses with broad neutralizing activity against SARS‐CoV‐2 variants, offering a promising candidate for next‐generation COVID‐19 vaccines. Another Chinese team developed a lymph node‐targeting circRNA‐mannose‐modified LNP vaccine platform, successfully applied to rabies virus and COVID‐19 prevention, eliciting strong immune responses.^223^ For other diseases, our team utilized circRNA to express the CMV‐pp65 antigen in monocyte‐derived DCs (moDCs), demonstrating that circRNA encoding the antigen can provide a more sustained antigen signal compared with mRNA, leading to more efficient activation and expansion of antigen‐specific T cells.^111^ The team led by Zhou et al.^199^ successfully developed a circRNA monkeypox vaccine targeting MPV antigens. This vaccine elicited robust humoral and cellular immune responses in mice and effectively protected immunized mice from MPV challenge.^199^ Professor Howard Y. Chang has also made substantial contributions to circRNA vaccine research, discovering that circRNA itself can act as an excellent vaccine adjuvant, stimulating innate immune responses and inducing long‐lasting protective antibodies in mice.^113^ Li et al.^198^ established an LNP‐circRNA vaccine platform, demonstrating the effectiveness of circRNA vaccines in tumor models with poor tumor immune microenvironments (TME). Moreover, they found that combining circRNA vaccines with adoptive cell therapy can enhance antitumor effects.^198^ The researchers developed a late‐stage immune desert orthotopic model and administered RNA vaccines, TCR‐T therapy, and a combination of both treatments. The results indicated that all tumors were completely eradicated in the combination group, suggesting that the circRNA vaccine combined with TCR‐T therapy is more effective than either treatment alone (Figure 5A).^198^

In conclusion, the intrinsic stability, prolonged protein expression, and innate adjuvant properties exhibited by circRNA present compelling advantages compared with mRNA. These distinctive characteristics position circRNA vaccines as highly promising candidates for future advancement in the field of immunization.

### 5.2 Gene expression modulation

Endogenous circRNA has been documented to intricately modulate gene expression through diverse mechanisms, such as serving as miRNA sponges, protein sponges, and outcompeting mRNA.^1^ Consequently, there is compelling rationale to anticipate that synthetic circRNA may likewise exert regulatory roles on specific target genes.

In 2022, two consecutive articles were published in the *Nature Biotechnology* journal, spotlighting the application of circRNA‐guided endogenous ADAR family‐mediated RNA editing technology.^170^, ^171^ Professor Wensheng Wei's team discovered that employing circ‐ADAR‐recruiting RNAs (arRNAs) instead of their linear counterparts resulted in a threefold increase in average editing efficiency, while maintaining effective editing for nearly two weeks. They further demonstrated that genetically encoded circ‐arRNA achieved long‐term RNA editing in human primary cells and organoids via adenoviral vector delivery.^170^ Concurrently, Katrekar et al.^171^ also found that utilizing circ‐arRNAs significantly enhanced RNA editing efficiency both in vivo and in vitro. In the realm of gene silencing, Zhang et al.^227^ employed a chemical synthesis method to produce circular siRNAs, revealing their superior long‐term gene silencing effects in vitro and in vivo, while minimizing off‐target gene silencing compared with conventional linear duplex siRNA molecules. Synthetic circRNA has also been harnessed as miRNA sponges. Lavenniah et al.^169^ developed a circular miRNA sponge targeting cardiac prohypertrophic miRNA‐132 and miRNA‐212, effectively sequestering these miRNAs and inhibiting their endogenous function, thereby mitigating pressure overload‐induced cardiac hypertrophy. Similarly, Wang et al.^229^ utilized an enzymatic ligation method to construct a circular miRNA sponge targeting miRNA‐21 and miRNA‐93, suppressing the proliferation of esophageal carcinoma cells and impeding tumor growth in murine xenograft models. Likewise, Liu et al.^228^ synthesized a circRNA sponge competitively inhibiting miRNA‐21, consequently suppressing the downstream proteins regulated by miRNA‐21. Additionally, circRNA can serve as protein sponges to regulate gene expression. Schreiner et al.^230^ developed a circRNA molecule designed to bind and inactivate heterogeneous nuclear ribonucleoprotein L, effectively modulating splicing‐regulatory networks in mammalian cells. Moreover, Lu et al.^41^ delivered circ‐INSR to cardiomyocytes and mouse models with cardiotoxicity, resulting in significant improvements in both cellular and murine conditions. The cardioprotective effects of circ‐INSR were attributed to its interaction with the single‐stranded DNA‐binding protein.^41^ Chen et al.^231^ optimized RNA self‐circularization to synthesize a low‐immunogenicity circRNA aptamer. They have elucidated its molecular mechanism in inhibiting PKR activation. Additionally, they established a mouse model overexpressing this circRNA aptamer, confirming its safety in vivo.^231^ Furthermore, targeted delivery of the circRNA aptamer to the spleen enabled intervention therapy in a mouse model of psoriasis associated with aberrant PKR activation.^231^

Recent breakthroughs in circRNA research highlight its expanding significance in RNA editing and gene regulation. Looking ahead, ongoing exploration of circRNA's diverse roles in gene modulation offers substantial potential for advancing precision medicine and tackling intricate health issues (Figure 5B).

### 5.3 Adoptive cell therapy

In the realm of adoptive cell therapy, traditional methods often entail ex vivo viral gene editing and transduction to engineer immune cells targeting specific tumor antigens.^232^ However, the intricate gene editing process and personalized nature of therapeutic products limit their widespread clinical use. In response, circRNA‐based in vivo/in situ adoptive cell therapy has emerged as a promising alternative, offering safer and more efficient treatment without the need for cumbersome ex vivo preparation.^233^, ^234^ Notably, oRNA Therapeutics has pioneered a groundbreaking synthetic circRNA combined with an innovative immunotropic LNPs platform, enabling efficient delivery of chimeric antigen receptor (CAR) circRNA to immune cells in preclinical models.^116^ This approach demonstrates potent and sustained cytotoxicity and cytokine production in anti‐human CD19 CAR‐T cells, surpassing control groups.^116^ Our team has generated pp56‐TCR‐T cells using pp56‐TCR circRNA with electroporation.^111^ In vivo experimental results demonstrated that a single infusion of circRNA‐pp65‐TCR‐T cells every two weeks effectively eliminated target cells expressing CMV‐pp65 antigen^111^ (Figure 5C).

The main benefit of circRNA‐based CAR‐T/TCR‐T construction is its transient expression of CAR/TCR protein in target cells without altering the host genome, reducing the potential for long‐term side effects. Additionally, circRNA‐based CAR‐T/TCR‐T offers a shift from highly personalized to widely applicable therapeutic options. Finally, its lower treatment cost compared with current virus‐based CAR‐T therapies adds to its appeal as a cost‐effective treatment alternative. However, the transient expression of CAR structures may exacerbate the difficulties of CAR‐T cell therapy in treating solid tumors. Therefore, combining CAR‐T with other therapies holds promise for overcoming the current challenges. Ma et al.^235^ demonstrated that administering a vaccine carrying the same antigen as CAR‐T cells shortly after injecting these cells into mice significantly enhanced CAR‐T cell efficacy against glioblastoma. This “vaccine + CAR‐T” combination not only bolstered the engineered CAR‐T cells but also promoted the generation of host T cells targeting other tumor antigens, a process known as “antigen spreading.”^235^, ^236^ This resulted in a robust and diverse T cell response, overcoming tumor antigen heterogeneity, eradicating tumors, and preventing recurrence.^236^ Sahin and his team^237^ from the BioNTech Therapeutics reported phase 1/2 clinical data on the treatment of Claudin 6 (CLDN6)‐positive relapsed or refractory advanced solid tumors. They employed CAR‐T cells targeting the CLDN6 antigen, with or without the addition of an mRNA vaccine encoding the CLDN6 antigen (CARVac).^237^ In a cohort of 21 patients analyzed for efficacy, the CLDN6 CAR‐T cell therapy, either alone or in combination with the CARVac, achieved an objective response rate (ORR) of 33% and a disease control rate of 67% in treating solid tumors.^237^ Notably, the ORR for some germ cell tumor patients reached 57%.^237^ This study marks the first instance of using an mRNA vaccine to enhance CAR‐T cell functionality, demonstrating the absolute superiority of the combined treatment approach. The aforementioned findings prompt an intriguing question: Could the concurrent delivery of circRNA encoding CAR constructs and circRNA encoding vaccines targeting the same antigen into the body result in synergistic therapeutic effects? Recently, Wang et al.^224^ published a study on bioRxiv in which they identified immune‐tropic LNPs used to deliver circRNAs encoding anti‐HER2 CAR structures and HER2 antigens. The anti‐HER2 CAR circRNA‐LNPs were administered intravenously to a colorectal cancer mouse model, while the HER2 antigen circRNA‐LNPs were administered intramuscularly.^224^ The study compared survival rates and tumor reduction among groups treated with PBS, CAR‐T cells alone, vaccine alone, and the combination of CAR‐T cells and the vaccine. The combination therapy group showed the most significant tumor reduction and extended survival.^224^ Our team is currently developing a circRNA‐LNP‐based anti‐DLL3 in vivo CAR‐T therapy. Preliminary results indicate an in vivo CAR‐T positive rate of approximately 15%. Additionally, we have purified DLL3 antigen protein and plan to combine the in vivo CAR‐T therapy with traditional protein vaccines to further enhance the antitumor effects (data not published). Undoubtedly, combination therapy will be a significant trend in the future of circRNA‐based treatments. Due to the heterogeneity of tumors, single therapies often fail to provide a curative or sustained effective treatment for patients. Combining circRNA therapies with other treatment modalities can significantly improve patient symptoms and prolong survival. Therefore, more exploration and development are required in the field of circRNA combination therapies.

### 5.4 Other

In other applications, harnessing the protein translation capabilities of circRNA enables precise and localized protein expression within specific cellular compartments or tissues, facilitating targeted modulation of protein function in a spatiotemporal manner. For instance, Yang et al.^114^ developed a circRNA encoding a variety of cytokines including active IL‐15, IL‐12 single chain, granulocyte macrophage colony‐stimulating factor, and IFN‐α 2b. By directly administering the circRNA into tumors, they effectively modulated the TME and notably enhanced the effectiveness of immunotherapy.^114^ In their experiment, circRNA was administered intratumorally every three days, with or without intraperitoneal injection of anti‐PD‐1 antibodies. The results demonstrated significant tumor reduction and prolonged survival in mice receiving the combination treatment, indicating that the combined therapy is markedly more effective than monotherapy.^114^ Xu et al.^200^ employed combinatorial chemistry techniques to synthesize customized LNPs tailored for lung cancer. In a Lewis lung cancer model, respiratory delivery of circRNA encoding interleukin‐12 efficiently induced robust immune responses, leading to pronounced tumor regression.^200^ Our research team has also developed a circRNA encoding IL‐12 (cmRNA1210), which, when delivered via LNP, demonstrates the ability to achieve an abscopal effect and inhibit the progression of lung metastases in both syngeneic mouse tumor models and humanized mouse tumor models.^225^ Moreover, combining cmRNA1210 with a checkpoint inhibitor results in a synergistic effect that significantly reduces the rate of tumor relapse.^225^ Recently, cmRNA1210 for solid tumor therapy has been evaluated for pre‐Investigational New Drug (IND) in China National Medical Products Administration. A Chinese research team utilized a ribozyme‐based method to engineer circRNA that encode spider silk proteins.^238^ This innovative approach has the potential to expedite the advancement of fibrous protein‐based biomaterials.^238^ Our research team has developed RiboPROTAC, a circRNA encoding proteolysis targeting chimera.^226^ This groundbreaking technology leverages the ubiquitin‐proteasome system to selectively degrade proteins, presenting a promising avenue for targeting previously undruggable proteins.^239^ Compared with traditional protein PROTACs, RiboPROTACs offer distinct advantages such as simplified cellular uptake and entry processes.^226^ With its potential to target undruggable protein targets, RiboPROTAC emerges as a novel and superior approach in the field. Another notable application is protein replacement therapy, a field where mRNA is extensively utilized.^240^, ^241^ This approach optimally restores tissue and cellular function by introducing mRNA encoding proteins that are lacking or defective due to genetic mutations into specific cell tissues. While mRNA has been widely explored in this context, circRNA also holds theoretical potential for use in protein replacement therapy, either as a substitute for or enhancement to mRNA. Recently, our team synthesized a proprietary ionizable glycerolipid with branched tails and five ester bonds, named TG6A, through a three‐step esterification process.^201^ LNP constructed with this lipid successfully transfected mesenchymal stem cells with circRNA encoding the FGF18 protein, which enhances cartilage regeneration.^201^ This significantly promoted cartilage repair in a rat osteoarthritis model, showing promise as a potential therapeutic candidate for curing osteoarthritis.^201^ In addition, our research team has successfully utilized LNP delivery to introduce circRNA encoding PTEN into osimertinib‐resistant lung cancer cells, resulting in the reversal of their drug resistance (data not published). However, research on protein replacement therapy utilizing circRNA remains limited at present (Figure 5D).

## 6 CONCLUSIONS AND PERSPECTIVES

Synthetic circRNA therapeutics have emerged as a promising field with the potential to revolutionize RNA‐based therapies. The synthesis of circRNA can be achieved through diverse methods including chemical synthesis, T4 RNA ligase synthesis and ribozyme synthesis. During the synthetic process, the precise design and synthesis of circRNA offer unprecedented control over their structure and function, enabling personalized medicine approaches and targeted treatments. Optimization strategies have been explored to enhance circRNA's protein expression capacity, minimize its immunogenicity and improve its stability. These factors are crucial for achieving therapeutic efficacy and ensuring the safety of circRNA‐based therapies. In addition, several delivery vectors, including viral vector, electroporation, LNP and exosome, are widely applied in circRNA therapeutics. Moreover, the versatile nature of synthetic circRNA allows for a wide range of applications, which spans across diverse areas including vaccines, gene regulation, adoptive cell therapy and more. It holds promise as a vaccine platform against infectious diseases and cancer. Its ability to regulate gene expression provides opportunities for the treatment of various disorders.

To date, no synthetic circRNA therapies have entered clinical trials or received regulatory approval, indicating that the development and application of circRNA therapies still face significant challenges. In future, translating circRNA therapies from bench to bedside involves several crucial steps:

1. *Manufacturing*: developing scalable and reproducible manufacturing processes for circRNA to ensure high purity, stability, and quality. Developing efficient and high‐yield purification methods for circRNA, with a focus on removing contaminants, is essential for scaling up circRNA production in industry. Additionally, further exploration into the factors influencing circRNA immunogenicity and the development of strategies to mitigate immune responses will enhance the safety and efficacy of circRNA therapeutics.
2. *Optimization and formulation*: optimizing delivery systems, such as LNPs, to ensure efficient targeting of circRNA to specific cells or tissues, while maintaining stability in biological environments, is crucial. Advancing delivery strategies tailored for circRNA, including refining associated LNP formulations and minimizing off‐target effects and toxicity of LNP is necessary for enhancing the biosafety of circRNA‐based therapies.
3. *Preclinical research*: conducting extensive in vitro and in vivo studies to evaluate the efficacy and safety of circRNA therapeutics, followed by toxicology assessments and immunogenicity testing. Comprehensive evaluation of the pharmacokinetics and long‐term effects of circRNA therapeutics is necessary for successful clinical translation.
4. *Regulatory approvals*: submitting an IND application to regulatory authorities (such as the US FDA) to gain approval for starting clinical trials. Synthetic circRNA therapeutics encounter significant regulatory challenges, including rigorous safety evaluations to detect potential immunogenicity and toxicity. Compliance with Good Manufacturing Practices is critical to ensure production consistency and product integrity. Efficacy must be demonstrated through well‐designed clinical trials, while intellectual property considerations and adherence to relevant legal and ethical standards are also crucial. Additionally, comprehensive long‐term impact assessments are required to monitor and address potential adverse effects. These regulatory requirements are essential to ensure the safety and effectiveness of circRNA therapeutics.
5. *Clinical trial design*: designing robust clinical trials with well‐defined endpoints, control groups, and appropriate patient populations to evaluate the therapeutic potential of circRNA.
6. *Patient monitoring*: implementing rigorous patient monitoring protocols to track therapeutic outcomes, manage potential side effects, and gather real‐world evidence on the safety and efficacy of circRNA therapies;
7. *Collaboration*: fostering collaboration between academic researchers, industry partners, and regulatory agencies to accelerate the translation process and address scientific and regulatory challenges.

In summary, synthetic circRNA therapeutics offer exciting prospects for RNA‐based therapies. By optimizing synthesis methods, enhancing functional attributes and exploring diverse applications, synthetic circRNA has the potential to revolutionize personalized medicine and address unmet clinical needs. Continued multidisciplinary research efforts are essential to fully unlock the potential of synthetic circRNA therapeutics and translate them into transformative treatments.

## AUTHOR CONTRIBUTIONS

*Conception and design*: Mantang Qiu and Chijian Zuo. *Administrative support*: Kezhong Chen and Yun Li. *Provision of study materials*: Jingsheng Cai, William Chi‐Shing Cho, Zheng Liu, Shaoyi Chen, and Haoran Li. *Manuscript writing*: Jingsheng Cai and Zonghao Qiu. *Final approval of manuscript*: all authors.

## CONFLICT OF INTEREST STATEMENT

Author Zonghao Qiu and Chijian Zuo are employees in Suzhou CureMed Biopharma Technology Co., Ltd. The other authors have no conflicts of interest to declare.

## ETHICS STATEMENT

Not applicable.

## Data Availability

Not applicable.
